# Office paper decorated with silver nanostars - an alternative cost effective platform for trace analyte detection by SERS

**DOI:** 10.1038/s41598-017-02484-8

**Published:** 2017-05-30

**Authors:** Maria João Oliveira, Pedro Quaresma, Miguel Peixoto de Almeida, Andreia Araújo, Eulália Pereira, Elvira Fortunato, Rodrigo Martins, Ricardo Franco, Hugo Águas

**Affiliations:** 1 i3n/CENIMAT, Departamento de Ciência dos Materiais, Faculdade de Ciências e Tecnologia, Universidade NOVA de Lisboa and CEMOP/UNINOVA, Campus de Caparica, 2829-516 Caparica, Portugal; 2 REQUIMTE/UCIBIO, Departamento de Química, Faculdade de Ciências e Tecnologia, Universidade NOVA de Lisboa, 2829-516 Caparica, Portugal; 3 LAQV-REQUIMTE, Departamento de Química e Bioquímica, Faculdade de Ciências, Universidade do Porto, 4169-007 Porto, Portugal

## Abstract

For analytical applications in portable sensors to be used in the point-of-need, low-cost SERS substrates using paper as a base, are an alternative. In this work, SERS substrates were produced on two different types of paper: a high porosity paper (Whatman no. 1); and a low porosity paper (commercially available office paper, Portucel Soporcel). Solutions containing spherical silver nanoparticles (AgNPs) and silver nanostars (AgNSs) were separately drop-casted on hydrophilic wells patterned on the papers. The porosity of the paper was found to play a determinant role on the AgNP and AgNS distribution along the paper fibres, with most of the nanoparticles being retained at the illuminated surface of the office paper substrate. The highest SERS enhancements were obtained for the office paper substrate, with deposited AgNSs. A limit of detection for rhodamine-6G as low as 11.4 ± 0.2 pg could be achieved, with an analytical enhancement factor of ≈10^7^ for this specific analyte. The well patterning technique allowed good signal uniformity (RSD of 1.7%). Besides, these SERS substrates remained stable after 5 weeks of storage (RSD of 7.3%). Paper-induced aggregation of AgNPs was found to be a viable alternative to the classical salt-induced aggregation, to obtain a highly sensitive SERS substrates.

## Introduction

Surface-enhanced Raman spectroscopy (SERS) has been intensively studied as a non-destructive and sensitive technique for applications in molecular detection, combining the specificity of vibrational Raman spectroscopy, with the increased sensitivity provided by plasmon assisted scattering, induced by metal nanostructures^1^. Due to the intense electromagnetic and chemical enhancements from SERS substrates-analyte, single molecule identification, under specific conditions, has been demonstrated in SERS, surpassing the weak effect of Raman scattering^2, 3^. Thus, significant efforts have been made in the development of reliable, portable and highly sensitive SERS platforms for point-of-need applications^4–12^. These attempts allowed SERS to rapidly emerge as a reliable and fast route in a diversity of fields, branching out into the detection of analytes in trace concentrations such as chemical detection^13^, reaction dynamics^14^, biosensing^15, 16^, explosives detection^17^, and art conservation^18^.

Although SERS has the potential to be a highly sensitive, rapid and portable analytical technique, the reproducible fabrication of highly active and uniformly enhancing SERS substrates still hinders the point-of-need application of SERS^1, 8^. SERS-active surfaces have been fabricated by several innovative techniques, including nanolithography^19, 20^, template-based^21^, self-assembly^22^ and several deposition techniques for colloidal metal nanoparticles^23, 24^. Though many of these approaches provide substrates for SERS with excellent enhancement factors (EF) values, several problems related to poor reproducibility and uniformity still need to be addressed. To improve these parameters, the optimization of SERS substrates has been pursued both by improving the enhancing properties of the materials used in their fabrication, and by refining the fabrication methods.

Gold and silver colloidal nanoparticles are commonly employed to prepare SERS substrates due to their simple and green synthesis, stability and favourable enhancement properties. EF values obtained for analytes adsorbed to gold and silver nanoparticles can be tuned by a variety of parameters such as particle size, shape, interparticle spacing, as well as changes in environment^25^. For instance, for spherical nanoparticles, aggregation (by centrifugation^26^ or salt addition^27^) has been used to improve the SERS signal, since the interstitial gaps formed between nanoparticles are *hot spots, i.e*. zones of particularly strong signal enhancement of entrapped analytes (the electric field is significantly improved)^28^. Because nanoparticle’s aggregation is a dynamic process in which the nanoparticle size distribution changes over time, this method is difficult to control and presents a low reproducibility^8^.

Another promising approach is the use of anisotropic metal nanoparticles, especially metal nanoparticles with shapes comprising sharp tips, that originate extremely strong *hot spots* that do not require aggregation methods^29, 30^. In particular, star-shaped nanoparticles have a high number of *hot spots per* particle, and surface plasmon resonance (SPR) extended over a large range of wavelengths, both useful characteristics for SERS applications in biodiagnostics and chemical detection. Recently, Garcia-Leis *et al*. reported the synthesis of star-shaped silver nanoparticles without strong capping agents or surfactants that could inhibit the adsorption of analyte and thus their use on SERS-based detection methods^23^.

A re-emerging platform for SERS substrates is cellulose paper (first described by Tran^31^ in 1984 and renewed interest in the more recent years) and comparable with the conventional rigid and planar supports such as glass^32^, silicon wafer^32^ and aluminium films^21^. Paper, however, has the advantage of being recyclable, biodegradable, and originated from an abundant renewable source^8^. In fact, paper-based SERS devices exploit the striking features of paper such as: (1) wicking capability (due to capillary forces); (2) flexibility and thinness (useful for swabbing, impossible for rigid platforms); (3) concentration of analytes trough lateral-flow paper microfluidics; (4) porosity and (5) sample storage capability^33, 34^. Hence, paper substrates are considered well suited for resource-limited countries in several areas of medical diagnostics^35, 36^, food analysis^37^, and environmental research^33, 36, 38–44^. To fabricate SERS substrates, metal nanoparticles have been attached to paper via soaking^45^, inkjet printing^8, 46, 47^, screen printing^5, 8, 11^, deposition^48^, filtration or *in-situ* growth^49^. These variety of methods led to paper SERS substrates with enhancement factors of about 10^5^–10^7^, which is equivalent to many of the more expensive and complex self-assembly and directed assembly techniques^8^.

In this work, paper SERS substrates were fabricated using anisotropic silver nanostars (AgNSs) for increased sensibility. For comparison, spherical nanoparticles (AgNPs) were also used, but in this case SERS EF values were one order of magnitude lower. The fabrication of paper SERS substrates involved the so called *Lab-on-paper* technology, including the printing of hydrophobic wax patterns and barriers on paper^33^. These patterns were then advantageously used to support the metal nanoparticles deposited by drop-casting method. We have evaluated the use of filter paper (Whatman no. 1), and standard white office paper. The latter has a lower porosity and is less hydrophilic with better ink retaining for writing and printing works^50^, a characteristic that was explored to obtain a more uniform distribution of the nanoparticles at the paper surface and higher EF values. Rhodamine 6 G (R6G), has been selected as a SERS probe because of its very intense and distinct Raman signals^51, 52^. By using silver nanoparticles-embedded office paper-SERS substrates, we could identify R6G in the ppb range. In addition, we have shown the signal uniformity, its reproducibility across different batches of AgNSs, and its time-stability over five weeks.

## Experimental section

### Chemical Synthesis

Spherical and star-shaped nanoparticles were synthesized by a chemical reduction method. All glassware was rinsed with acetone, followed by Milli-Q water, before use. All chemicals were used without further purification or modification. Milli-Q water (purified by a Millipore system - *Millipore Corporation*) 18.2 MΩ·cm^−1^ was used throughout the syntheses and experiments.

#### Synthesis of AgNPs

Citrate capped spherical silver nanoparticles (AgNPs), were prepared based on the method of Bastús *et al*.^53^. Briefly, 100 mL of an aqueous solution containing sodium citrate tribasic dihydrate (5 × 10^−3^ M) (Sigma-Aldrich, 99%) and tannic acid (10^−4^ M) (Sigma-Aldrich, 95%) was heated under reflux over vigorous stirring. When the boiling started (≈15 min), 1 mL of AgNO_3_ (Sigma-Aldrich, 99.8%) was rapidly added and the solution immediately became bright yellow. The solution was kept under heating and stirring for 5 min and then cooled with ice. In order to remove the excess of tannic acid from the resultant coated AgNPs, the solution was purified by centrifugation (Eppendorf) at 12 000 *g* for 25 min and with a temperature of 4 °C, and redispersed in sodium citrate (2.2 × 10^−3^ M) before sample characterization by UV-Vis absorption spectroscopy. The final colloidal solution of AgNPs presented a LSPR band centred at approximately 400 nm. The average diameter and molar concentration of the AgNPs was estimated by visible spectrophotometry according to the methodology proposed by Paramelle *et al*.^54^. This method is based on previous work of Haiss *et al*.^55^, for spherical AuNPs capped with citrate. For spherical AgNPs the average diameter is estimated by this method and confirmed by TEM. Finally, the concentration of AgNPs is obtained by a Beer-Lambert equation, using the extinction molar coefficient calculated for AgNPs with that average diameter^54^. Further details are presented in Supplementary Information – section S1. The several concentrations used throughout the work were achieved by centrifugation at 12 000 *g* for 20 min and with a temperature of 4 °C and confirmed by Paramelle *et al*. method^54^.

#### Synthesis of AgNSs

The procedure for the production of citrate capped star-shaped silver nanoparticles, or silver nanostars (AgNSs), was recently reported by Garcia-Leis *et al*.^23^. Briefly, a solution containing 2.5 mL of hydroxylamine (60 × 10^−2 ^M) (Sigma-Aldrich, 50 wt.% in H_2_O) and 2.5 mL of NaOH (50 × 10^−2^ M) (Sigma-Aldrich, 98%) was prepared and placed under stirring, followed by dropwise addition of 45 mL of AgNO_3_ (10^−3 ^M) (Sigma-Aldrich, 99.8%). The colloidal solution changes form colourless to brown. After 2 min, 500 μL of sodium citrate (1.5%) (Sigma-Aldrich, 99%) was added to the mixture, and the dark grey solution was stirred for 3 h. After concentration by centrifugation (Eppendorf) at 1500 g for 15 min and resuspension in Milli-Q water, the colloidal solution was stored at 4 °C, in the dark, until use^23^. The hydrodynamic diameter and concentration of AgNSs were determined by Nanoparticle Tracking Analysis (NTA). In this method, the Brownian motion of the particles in a sample is recorded in video, using the light scattered by the nanoparticles to detect each individual particle. The measurements were performed using a NanoSight NS300 instrument in 1:50 diluted solutions in 1 mM sodium citrate, sonicated for 5 minutes before analysis. A flow cell was used, recording 5 videos of 60 seconds each, at a volume flow rate of 3 μL/min. The camera was set to automatic mode to avoid user bias. Videos were analysed using NS300 NTA 3.2 software with a threshold setting of 10. After, video analyses were performed by the software, which follows the path of the recorded bright circular shapes, the mean squared displacement (MSD) could be found for each particle. From these MSD values, the diffusion coefficient (D) was determined and, the hydrodynamic radius (r) can be calculated using the Stokes-Einstein equation^56^. Simultaneously the software records the number of particles *per* frame which can be used to determine the concentration of AgNS present in the colloidal solution. It should be mentioned that for high scattering materials, like silver and gold, NTA has a limit of detection of *ca*. 10 nm, and since the particles are detected individually there is no significant bias for larger particles, as typical for techniques based in light scattering. The concentrations reported are thus the concentration of nanoparticles in solution with average diameters higher than 10 nm. For additional details please see Supplementary Information – section S2).

The several concentrations used throughout the work were achieved by centrifugation at 1500 g for 10 min and with a temperature of 4 °C.

### Plasmonic paper substrate fabrication

**Figure 1:**
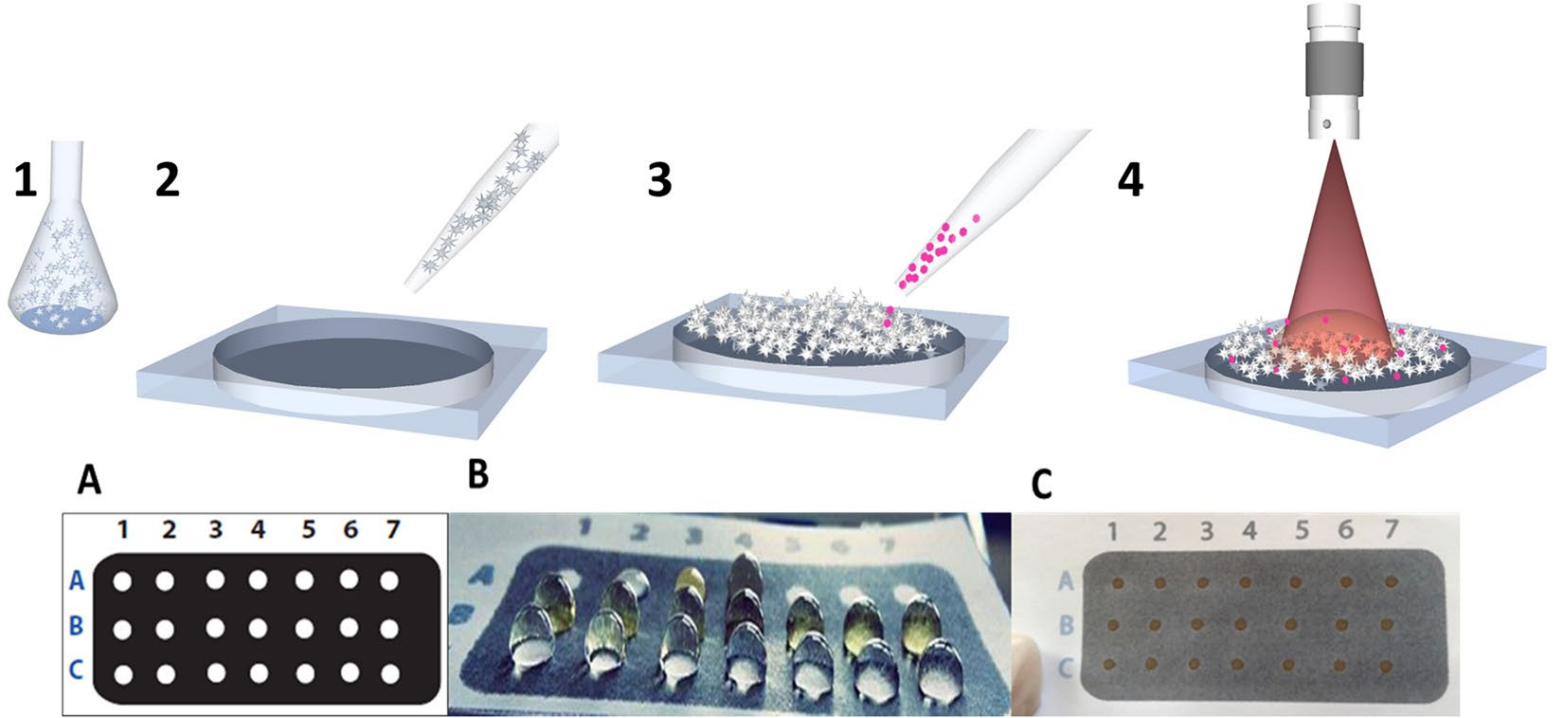
*Top:* Schematic representation of the fabrication process of the plasmonic SERS paper substrates: (1) Synthesis of (spherical and star-shaped) silver nanoparticles; (2) formation of wells on (Whatman no. 1 and office) paper followed by deposition of nanoparticles in the wells by drop-casting; (3) addition of R6G; (4) analysis of SERS signal using a 633 nm laser. *Bottom*: Wells pattern design on paper, before and after deposition of AgNSs. (**A**) Front view of the paper surface with wells presenting a diameter of 2 mm. (**B**) Photograph of the plasmonic paper substrate after drop-casting of the AgNSs solution and (**C**) after drying.

The two main stages for preparation of the plasmonic paper substrates were: (1) formation of wells by patterning of hydrophobic barriers (Fig. 1A); and (2) deposition of nanoparticles in the wells by drop-casting (Fig. 1B).

A pattern defining hydrophilic wells surrounded by hydrophobic barriers was printed in a Xerox ColorQube8570 (Xerox Corporation, Norwalk, CT, USA), a printer designed to use solid wax ink, on A5 (148 × 210 mm) and A4 (210 × 297 mm) sheets of Whatman no. 1 (Whatman Internacional Ltd., Florham Park, NJ) and office (300%, Portucel Soporcel, Setúbal, Portugal) papers, respectively. Well patterns were printed with diameters of 3 mm. The papers with printed patterns were placed on a hot plate (Heidolph MR HeiTec, Schwabach, Germany) at 140 °C for 2 min, allowing the wax to melt and diffuse vertically across the full thickness of the paper, creating the desired hydrophobic barrier^36^. After diffusion, the wells became smaller, with 2 mm of diameter (final diameter). The hydrophobic barriers, corresponding to the black areas in Fig. 1A are needed to confine the diffusion of the added solutions to the well. Diffusion outside the wells would result in a non-uniform distribution and a lower concentration of the nanoparticles such as previously reported by others (see Fig. S3 in Supplementary Information)^45, 57^. In addition, the barriers prevent contamination of adjacent samples, even in the more hydrophobic office paper^36^, allowing multiple assays.

AgNPs and AgNSs colloidal solutions (10 µL) were drop*-*casted in the wells forming the paper SERS substrate. The concentrations of AgNPs and AgNSs added to the 2 mm wells were 0.22, 0.44, 0.88, 1.76, 3.52, 7.04 nM. The several concentrations were achieved by centrifugation as mentioned above. The substrates were then gently dried by suspension on the top of a heating plate (Agimatic-N, Selecta). The final paper SERS substrates were stored at 4 °C in a desiccator, wrapped in aluminium foil. By knowing the concentrations and volumes dropped in the wells with a defined area it is possible to determine the corresponding amounts of nanoparticles *per* area of the well (NPs/mm^2^).

### Characterization

#### Morphological characterization

Scanning electron microscopy (SEM) observations of the plasmonic paper substrates were carried out in a Carl Zeiss AURIGA Crossbeam (FIB-SEM) Workstation equipped for EDS measurements. Transmission electron microscopy (TEM) specimens were prepared by placing one drop of the nanoparticles solution on a carbon-coated copper grid and drying at room temperature. TEM was performed with a HITACHI H-8100 microscope operated at 200 kV.

#### Optical characterization

All measurements in the UV-Vis spectrophotometer (Spectrophotometer Cary 50 Bio UV-Visible, Varian) of colloidal solutions were performed in a quartz cell with a path length of 1 cm (Hellma, Germany), with a wavelength range between 200 and 800 nm at room temperature. The optical response of the plasmonic paper substrates with AgNPs or AgNSs, was measured with a double beam UV-VIS-NIR spectrometer (Lambda 950, PerkinElmer) equipped with an integrating sphere, in the wavelength range of 300–1100 nm.

#### Raman measurements

Raman measurements were performed in a Labram 300 Horiba Jobin Yvon spectrometer equipped with an air-cooled CCD detector and a HeNe laser operating at 1750 µW of 632.81 nm laser excitation. The scattered light is passed through a solid edge filter (Horiba) for the removal of Rayleigh-scattered light in the region observed and provides a high throughput (90–95%) for Raman lines. The spectral resolution of the spectroscopic system is 4 cm^−1^ and the wavenumber for each point is 0.5 cm^−1^. The laser beam was focused with a 50 × Olympus objective lens (N10.6 LMPLAN FL N) with a numerical aperture of 0.75. An integration time of 5 scans of 25 s each, was used for all measurements; to reduce the random background noise induced by the detector, without significantly increasing the acquisition time. The experimental conditions allowed a minimization of the laser irradiation effects (optimum conditions tested previously)^58, 59^. To avoid the burning of the sample on the paper SERS substrates, the intensity of the incident laser was reduced to 660 µW on the surface of the sample by using suitable filtering. Triplicates were taken of all spectra. Between different Raman sessions, the spectrograph was calibrated using the Raman line at 521 cm^−1^ of a Si wafer for reducing possible fluctuations of the Raman system. All SERS spectra were recorded at room temperature.

Tetraethylrhodamine hydrochloride (Rhodamine 6 G – R6G) was purchased from Sigma-Aldrich, and used without further purification. SERS samples were prepared by dropping 2 μL of R6G solution on the plasmonic paper substrate. Concentrations of the R6G solution were in the range 10^−3^–10^−12 ^M, while a 10^−3 ^M R6G solution was used in glass as the reference control. Vibrational lines assignments for R6G are presented in Table S1 (see Supplementary Information). The areas of the vibrational Raman lines at 1360 cm^−1^ and 1509 cm^−1^ were used to calculate spectral intensity as usually reported in the literature^9, 11^. A limit of 200 a.u. was established as a threshold for a detectable SERS signal of R6G. Spectral analysis were performed with the curve-fitting program Peakfit v4.12 (Seasolve Software Inc.). After linear baseline subtraction, Lorentzian decomposition of spectra was performed to identify peak parameters (height, area, centre, and width) associated with selected Raman lines. The average SERS enhancement factor (EF) was calculated from the equation:

1 $$EF=\frac{{I}_{SERS}\times {N}_{Raman}}{{I}_{Raman}\times {N}_{SERS}}$$

where, *I*_*SERS*_ is the SERS intensity of a particular Raman line of the analyte (in this case R6G) and *I*_*Raman*_ is the normal (not enhanced) Raman intensity of the R6G measured over a non-plasmonic reference substrate (glass). *N*_*SERS*_ corresponds to the estimated number of molecules contributing to the SERS signal, while *N*_*Raman*_ is the number of molecules contributing to the reference Raman signal (from the non-SERS surface). For further details please see Supplementary Information – section S4 ^60^.

The signal of a Raman spectrum compared to a representative SERS spectrum of R6G is shown in Supplementary Information – section S5.

The average area of Raman lines at 1360 cm^−1^ and 1509 cm^−1^ were around 10^3^ a.u.

Uniformity of the R6G Raman signal in the wells was determined by performing a mapping with a micro-Raman, Renishaw inVia Reflex system using a step of 32.5 μm with a laser of 532 nm focused with a 50× Leica objective lens (N Plan EPI).

### Reproducibility and stability of plasmonic paper substrates

Replicates of the optimized wells were made to evaluate the reproducibility between different batches of AgNSs (n = 3). These substrates were tested with three different concentrations of R6G (10^−7^, 10^−8^ and 10^−9^ M). The SERS signal was also evaluated in each well, during 5 weeks using three different concentrations of R6G (10^−6^, 10^−8^ and 10^−9^ M). Between measurements, the paper SERS substrate was kept in the dark and stored at 4 °C in a desiccator.

## Results and Discussion

### Characterization of the plasmonic paper SERS substrates

**Figure 2:**
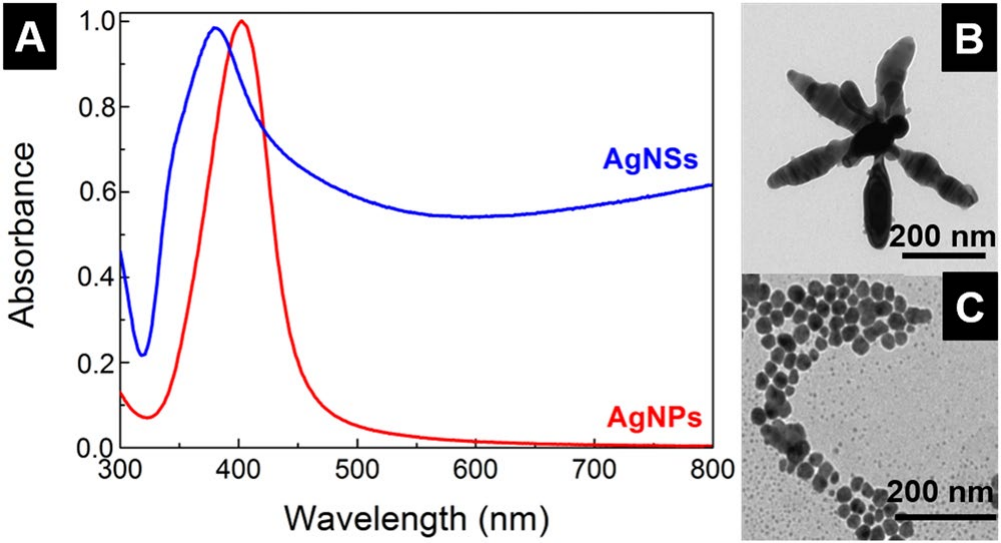
Characterization of AgNPs and AgNSs. (**A**) UV-vis spectra of non-aggregated spherical AgNPs (red) with λ_max_ = 402 nm; and AgNSs (blue) with λ_max_ = 379 nm. Transmission Electron Microscopy (TEM) micrographs showing a (**B**) magnification of a single AgNS; and (**C**) group of AgNPs.

Two types of nanoparticles were used: spherical silver nanoparticles (AgNPs) and silver nanostars (AgNSs). AgNPs used have an average diameter of ≈23 nm, and show the typical yellow colour corresponding to the plasmon band centred at around 400 nm (Fig. 2A) corresponding to the dipolar LSPRs of AgNPs which is in agreement with literature^25, 61^. Because the optical features strongly depend on shape and size of the nanoparticles is expected that the LSPR band is very different for spherical and for star-shape nanoparticles^62^. AgNSs are morphologically characterized by several tips protruding from a central core with a tip-to-tip length around 200 nm. The number of tips *per* nanoparticle varies between 7 and 12, with 8 being the average (Fig. 2B). The tips show an irregular morphology, with rounded borders and, in some cases, multiple branches. As typical for the synthesis of anisometric nanoparticles, other morphologies are detected in the samples, namely irregular spheres and rods, but these consistently represent less than 10% of the total nanoparticles, as evaluated by TEM analysis (see Supplementary Information, Fig. S1). The visible spectrum displays one strong plasmon resonance band at ≈379 nm that is usually attributed to quadrupole resonance, and a broad absorption extending to higher wavelengths arising from the multiple contributions of the AgNSs tips and the dispersion in morphology of the AgNSs, with different number of arms and varying tip sharpness being more associated to the dipole plasmon resonance (more details in Supplementary Information – section S7)^25^.

**Figure 3:**
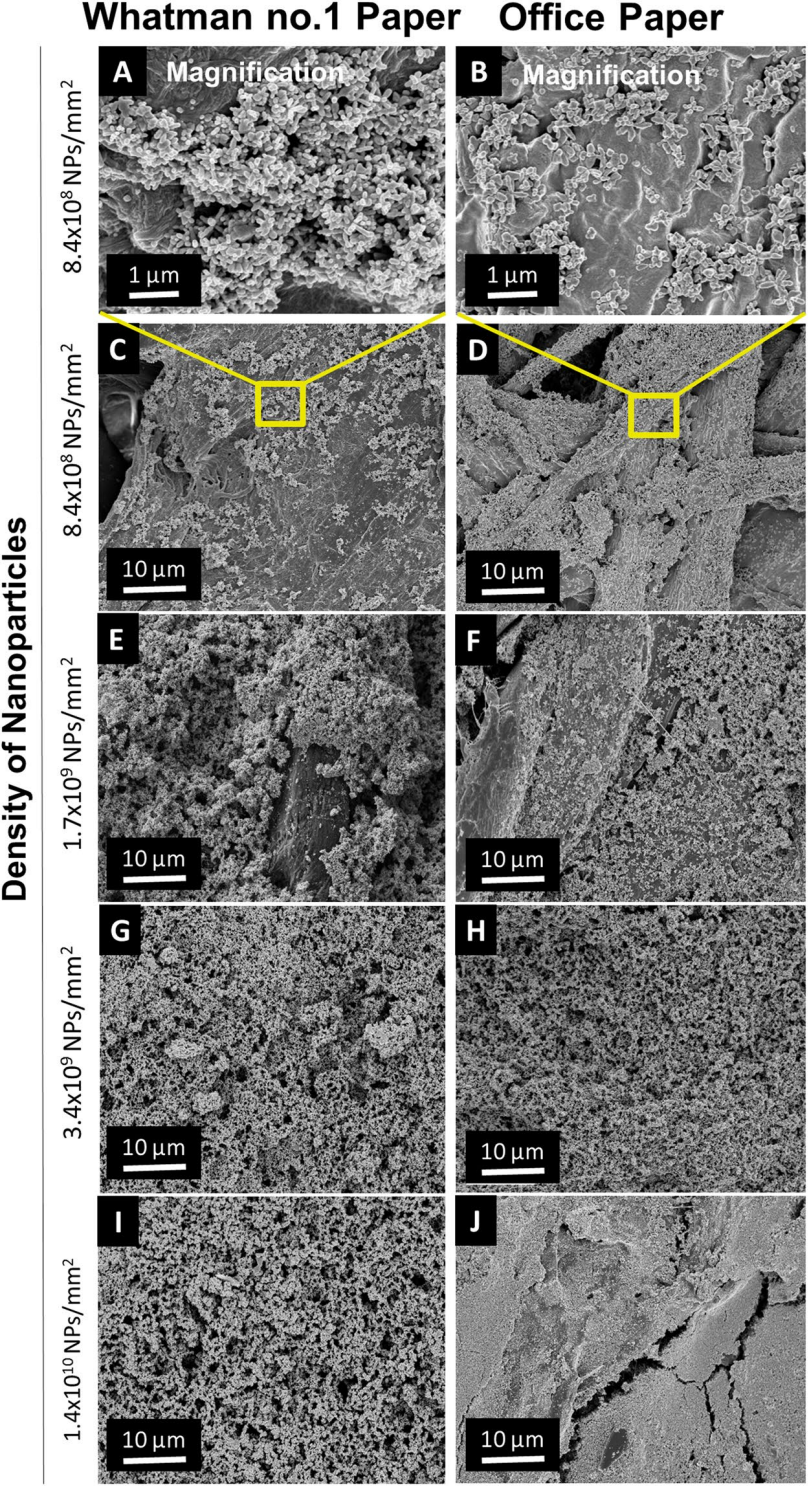
SEM micrographs of AgNSs in the two types of papers: Whatman no. 1 (**A,C,E,G**,**I**) and office paper (**B,D,F,H,J**). (**A** and **B**) are higher magnifications of (**C** and **D**), respectively with nanoparticle’s densities of 8.4 × 10^8^ NPs/mm^2^ showing the uniform distribution of AgNSs decorating the surface on Whatman no. 1 (**A**) and office (**B**) paper surfaces. From the top to the bottom of the figure, the amount of AgNSs added onto the paper well, increases from (**A**,**B**) and (**C**,**D**) 8.4 × 10^8^ NPs/mm^2^ (**E**,**F**) 1.7 × 10^9^ NPs/mm^2^ (**G**,**H**) 3.4 × 10^9^ NPs/mm;^2^ (**I**,**J**) 1.4 × 10^10^ NPs/mm^2^.

Figure 3 shows a SEM images of the SERS substrates fabricated in Whatman no. 1 and office papers after casting AgNSs at 0.4 nM (equivalent to 8.4 × 10^8^ NPs/mm^2^). In Fig. 3A,B the presence of AgNSs is clearly detected, but other nanoparticles with irregular shapes are also observed. These irregular nanoparticles were not detected in TEM characterization of the AgNSs synthesized, and are possibly artefacts due to the irregular surface of the paper matrix or to gold sputtering used in the preparation of the samples for SEM. A dense layer of nanoparticles can be observed for both papers; however, office paper provided a more uniform nanoparticle distribution without the formation of visible aggregates. The distribution for Whatman no. 1 paper is similar to other reports^6, 13^.

SEM analysis of the wells with increasing concentration of colloidal solution showed that, as expected, increasing the concentration of nanoparticles improves the coverage of the surfaces regardless of the type of paper (see also Fig. S5 in Supplementary Information). For the same number of applied nanoparticles, office paper shows a much larger amount of nanoparticles on the surface, when compared with Whatman no. 1 paper (see Fig. 3A,B). On Whatman no. 1 paper, it is necessary to deposit at least 3.4 × 10^9^ NPs/mm^2^ to obtain a closely packed assembly of nanoparticles, whereas on office paper, half the number of nanoparticles (1.7 × 10^9^ NPs/mm^2^) was enough to obtain the same effect.

**Figure 4:**
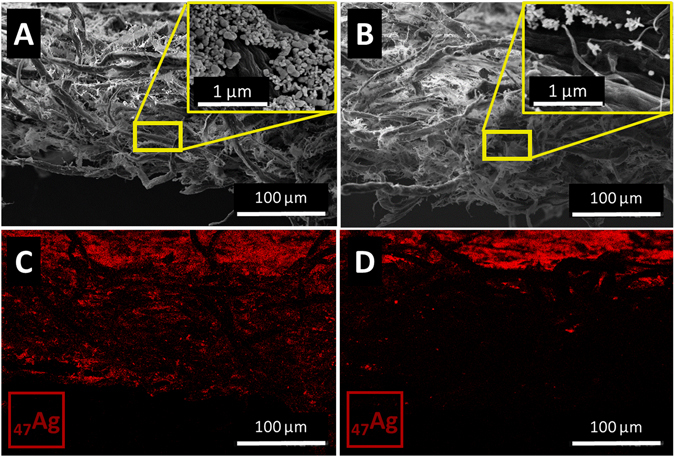
SEM micrographs and the respective EDS analysis images for elemental Ag, on the cross section of the paper plasmonic substrates with AgNSs on Whatman no. 1 paper (**A**,**C**), and on office paper (**B**,**D**). Silver can be detected across the bulk of the Whatman no. 1 paper although more concentrated at the surface; whereas for office paper, Ag is observed mostly at the substrate surface. Insets in (**A** and **B**) are magnified images of the bulk of the papers.

SEM-EDS analysis from a cross section of both papers exhibited a remarkably different Ag distribution map (Fig. 4). For Whatman no. 1 paper, AgNSs are concentrated at the surface but can be detected across the entire thickness of the well (Fig. 4A,C). This is in accordance with the results reported by Ngo *et al*. that estimated by SEM and UV-Vis that only 40% of AuNPs (≈23 nm) were adsorbed on the surface of the filter paper, while the remaining fraction (60%) appeared diffused within the bulk of the paper material^45^. Conversely, AgNSs added to office paper were mostly retained at the paper surface (Fig. 4B,D). These results are in agreement with the SEM results shown in Fig. 3, explaining why a lower quantity of nanoparticles is required for the office paper, compared to the Whatman no. 1, to achieve a uniform surface coverage of nanoparticles.

The uniform, irreversible adsorption of nanoparticles on the papers was possible by van der Waals interactions without the need for any chemical cross-linking procedures (Figs 3 and 4) such as reported elsewhere^6, 45^. The differences between the two types of paper used is likely related to their remarkably different porosity, being smaller for office paper (49%) than for Whatman no. 1 paper (68%)^36, 63^. This difference can account for the fact that nanoparticles are better retained and distributed more evenly on the surface of office paper, than on the Whatman no. 1 paper. Because Whatman no. 1 paper has a larger inherent heterogeneity, with pores and fibres of different sizes, the adsorption of nanoparticles was found to be less uniform. This effect is more obvious at lower colloidal concentrations. Therefore, the diffusion and adsorption of nanoparticles on paper seems to be mainly controlled by the three dimensional fibres network of paper that provides different strong capillary forces for the diffusion of nanoparticles from solution^45^.

**Figure 5:**
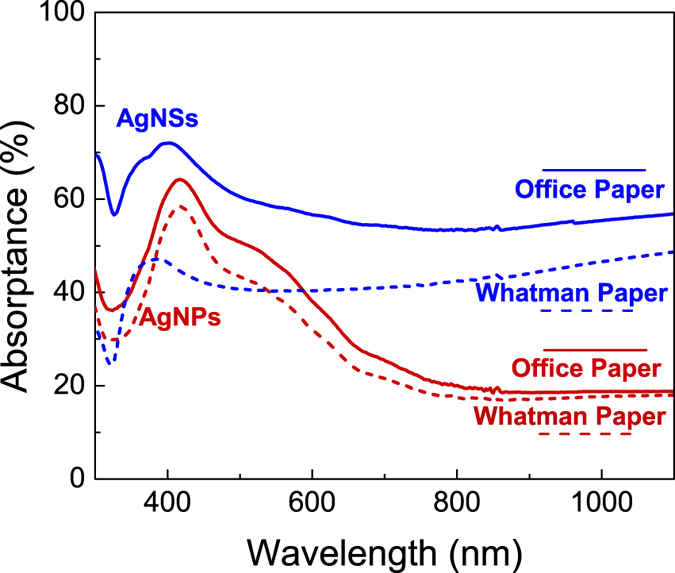
UV-VIS-NIR spectra of plasmonic paper substrates (Office paper – solid lines; and Whatman no. 1 paper – dashed lines), containing drop-cast AgNPs (red lines) or AgNSs (blue lines).

The light absorption spectra of AgNPs and AgNSs on paper are shown in Fig. 5. The spectra are very similar to the corresponding spectra of the nanoparticles in solution, except for a higher background absorption at higher wavelengths, possibly due to some aggregation on the paper. These background absorption is a consequence of the interaction between the electrical fields arising from all the particles^25^. The local absorption maximum due to plasmons is around 420 nm for the AgNPs and around 380 nm for AgNSs. It was expected that the absorptance in office paper would be lower due to the high reflectance of calcium carbonate present in this paper. However, the office paper is fabricated with CaCO_3_ and kaolin pigment particles specifically to fill and form a top coating, reducing its porosity^50^. Hence, as already demonstrated by SEM-EDS analysis, office paper has a higher number of nanoparticles retained on the surface, which leads to a lower paper reflectance and a higher absorptance from the nanoparticles, contrary to the Whatman no. 1 paper that do not have CaCO_3_ and kaolin pigment particles.

### Characterization of SERS performance

Paper-derived fluorescence is a concern when paper is used as substrate for SERS analysis^64, 65^. In fact, office paper samples showed fluorescence emission with excitation under the Raman laser (red beam, 633 nm). Interestingly, this fluorescence was eliminated after the addition of silver nanoparticles (see Fig. S6 in Supplementary Information). This can be explained by the fact that in the one hand, metal nanoparticles tend to quench fluorescence emission signals, by a Förster resonance energy transfer (FRET) mechanism. FRET is a distance-dependent process in which energy is transferred from an excited donor to an acceptor molecule that then emits lower energy light. On the other hand, the deposited metal nanoparticles on the surface can shield any fluorescence emission from the paper material^66^.

**Figure 6:**
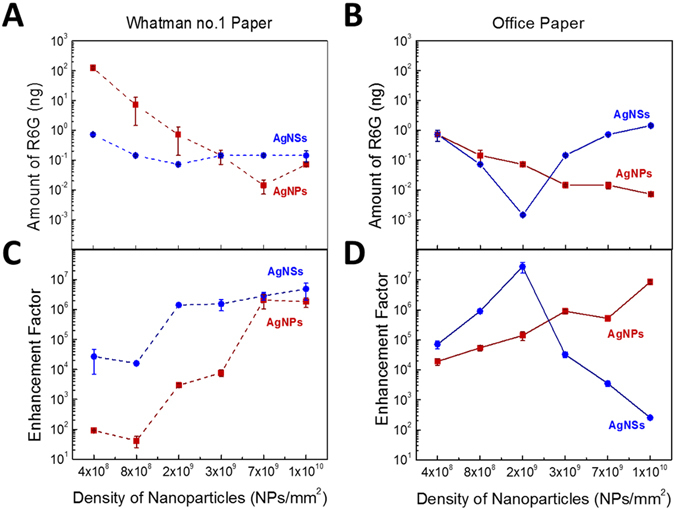
*Top*: Minimum amount of R6G (in ng) that can be detected on plasmonic substrates prepared on (**A**) Whatman no. 1 paper; and (**B**) office paper. Nanoparticle’ density was calculated by dividing the number of nanoparticles deposited by the area of the well. Data points from AgNPs- and AgNSs-wells are represented by red squares and blue circles, respectively. *Bottom:* Average EF values for R6G on (**C**) Whatman no. 1 and (**D**) office paper, with several nanoparticles densities. Average EF values were calculated from the 1509 cm^−1^ SERS vibrational line. Lines are guides to the eye.

The performance of the paper plasmonic substrates was evaluated by SERS of R6G, using different densities of AgNPs and AgNSs on the paper-wells (from 4.2 × 10^8^ up to 1.4 × 10^10^ NPs/mm^2^). Figure 6A,B, presents the minimum amount of R6G that could be detected by SERS (corresponding to an intensity of at least 200 a.u.) in office and Whatman no. 1 paper wells, loaded with different nanoparticle densities. Figure 6C,D, presents the average EF values at the corresponding detected minimum amount of R6G with the respective nanoparticle density. Raman and SERS spectra are in good agreement with respect to both the frequencies and the relative intensities of its vibrational lines, with those reported in the literature^67^. In fact, vibrational lines energies are highly conserved between Raman and SERS, indicating an electrostatic interaction between R6G and the Ag surface^67^. Raman and SERS spectra for R6G are presented and discussed further in the Supplementary Information – section S5. For AgNPs on both types of paper, smaller amounts of R6G can be detected with the increase of AgNPs density, with a lower limit of *ca*. 0.01 ng of R6G for the highest AgNPs densities. This lower limit was attained with a AgNPs density of 6.8 × 10^9^ NP/mm^2^ for Whatman no. 1 paper, whereas a lower AgNPs density of 1.7 × 10^9^ NP/mm^2^ was sufficient in the case of office paper (compare red data points and lines, on both panels of Fig. 6). For AgNPs on Whatman no. 1 paper, a stabilization of the average EF value was also obtained for AgNPs densities higher than 6.8 × 10^9^ NPs/mm^2^, corresponding to an average EF value of (2.1 ± 0.7) × 10^6^. Nevertheless, for lower AgNPs densities, average EF values were lower than those obtained with AgNSs. On office paper with AgNPs deposited, there was a monotonic increase of average EF values for higher nanoparticle’ densities, up to an average EF value equal to (8.4 ± 0.2) × 10^6^, for the maximum tested density of 1.4 × 10^10^ NPs/mm^2^.

The best result was obtained for AgNSs on office paper at a density of 1.7 × 10^9^ NPs/mm^2^, for which a minimum quantity of 0.001 ng (10^−9^ M) of R6G was achieved. The EF results closely follow the minimum detected amounts, with the highest average EF value (3.0 ± 0.7) × 10^7^ for AgNSs at a density of 1.7 × 10^9^ NPs/mm^2^ deposited on office paper, corresponding to the highest detection sensitivity. The sensitivity trends for AgNSs were very different from those observed for AgNPs. On Whatman no. 1 paper, the minimum amount of R6G detectable does not considerably change with the density of nanoparticles, with values in the range 0.1–1 ng for all AgNSs densities tested. The same AgNSs-Whatman no. 1 paper system, presented an increase of the average EF value with increasing AgNSs densities, stabilizing for densities higher than 1.7 × 10^9^ NPs/mm^2^, corresponding to an EF value of (1.41 ± 0.05) × 10^6^. Regarding the office paper, the minimum amount of R6G detectable shows a minimum at a density of 1.7 × 10^9^ NPs/mm^2^, followed by a decrease in detection sensitivity for the higher densities of nanoparticles, and consequently, lower average EF values.

It should be noted that the actual quantity of R6G being detected is much lower than the data presented in Fig. 6, since the Raman signal is being collected at the laser spot size with a diameter of ≈1 μm, while the quantity of nanoparticles indicated is the total amount deposited on the 2 mm-diameter well. Taking this geometrical factor into account, the minimum amount of R6G detected at the laser spot with AgNSs 1.7 × 10^9^ NPs/mm^2^-office paper system is in fact *ca*. 3 atto-grams, corresponding to only 3 × 10^3^ R6G molecules.

From the analysis of Fig. 6A,B, it is also apparent that office paper provides a more consistent pattern of enhanced detection over Whatman no. 1 paper, as assessed by the lower standard deviations for office paper than for Whatman no. 1. In fact, not only SERS spectra are noisier and less resolved in Whatman no. 1 paper, but also measurements were not consistent in all wells. A possible explanation for this behaviour is the more heterogeneous distribution of nanoparticles due the more porous structure of the Whatman no. 1 paper.

In office paper, it was possible to obtain reproducible SERS intensities with minimal noise, enabling a higher detection sensitivity than on Whatman no. 1 paper. However, for lower R6G amounts (0.1–0.001 ng *i.e*. 10^−7^–10^−9^ M), the spectra occasionally exhibited interferences. These interferences were attributed to the capping agents of the nanoparticles (see Supplementary Information – section S10 - for a more detailed discussion of this matter) and had no impact on SERS intensity measurements, as the area of the vibrational line at 1509 cm^−1^ was used to calculate the spectral intensity. This vibrational line appears in a region of the spectrum in which there is no interference from the capping agent vibrational lines, in contrast with the vibrational line at 1360 cm^−1^ ^59, 68^. The presence of interferences in paper SERS substrates underscores the importance of the interpretation of spectra profiles deriving from the substrate, even before the addition of the analyte.

**Figure 7:**
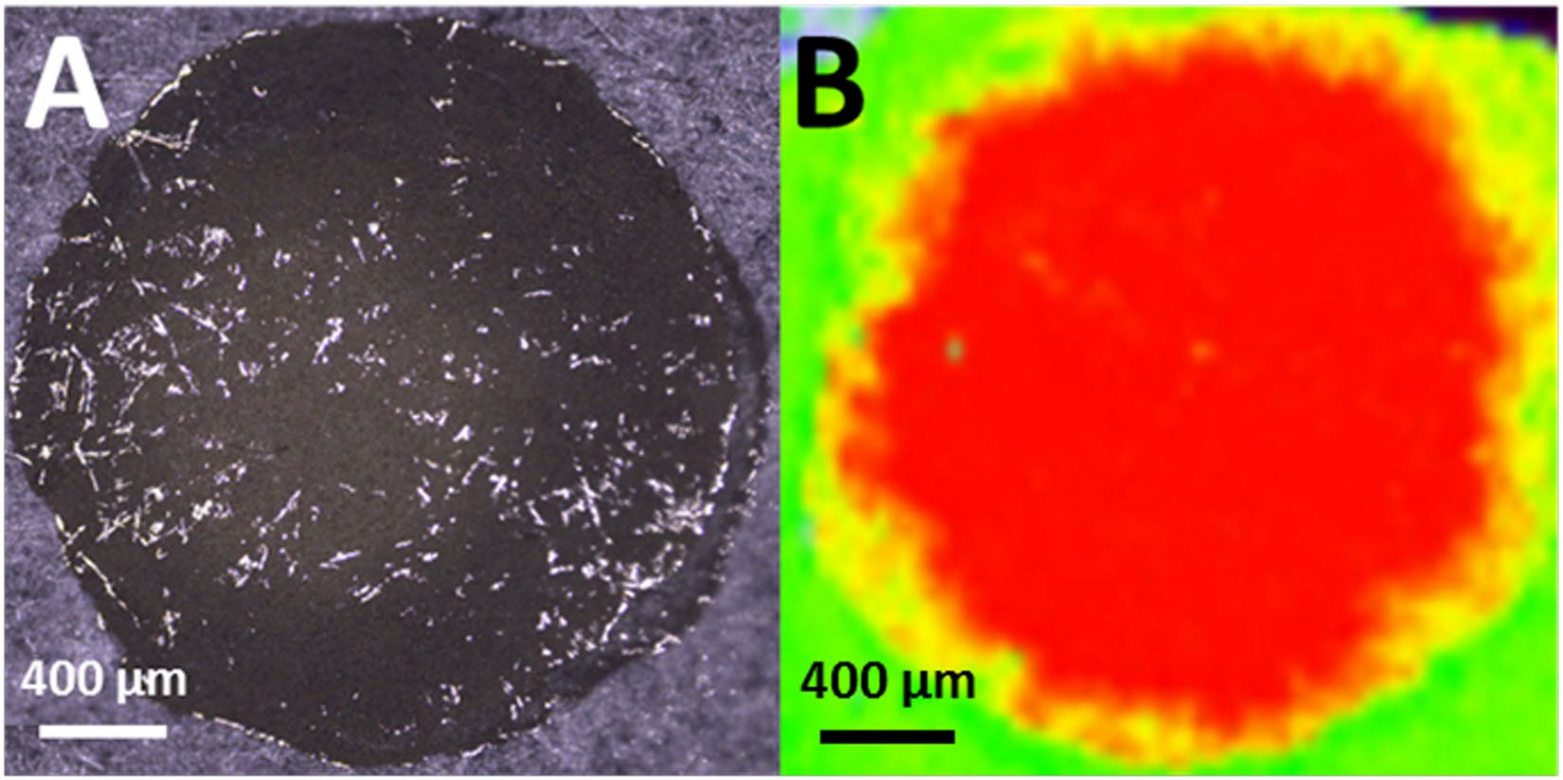
(**A**) Microscopic image of the SERS substrate on office paper. (**B**) Distribution of R6G molecules (red colour) across the entire area of the well in the plasmonic paper substrate obtained by Raman Imaging. Due to the extremely fast drying of the drop, R6G molecules are uniformly distributed on the well. The intensity of the red colour is proportional to the 1509 cm^−1^ vibrational line intensity from R6G. The green shows the absence of R6G signal.

The difference between the results obtained for Whatman no. 1 paper and office paper, namely, the greater SERS sensitivity of the latter, can be explained by the three-dimensional structure of the cellulose fibres. The larger capacity of office paper for retaining the drop-casted nanoparticles at the surface, favours the SERS effect, as a larger number of *hot spot* sites becomes available at the surface of the paper. Furthermore, when the R6G solution is added, the liquid diffuses into the paper and dries almost instantly due to the cellulose fibre structure (Fig. 7A,B). Hence, molecule adsorption is controlled by the swiftly drying of water, enabling a uniform distribution of nanoparticles and R6G on the paper surface. Because of the lower porosity of the office paper in relation to Whatman no. 1 paper, R6G molecules can be distributed uniformly on the surface, without any “coffee-ring” effect, promoting a high SERS signal uniformity (Fig. 7B). Thus, the porosity of the paper is a key-factor in the uniformity of the SERS signal obtained with these substrates. In addition, the relative Raman line intensities of the paper material (cellulose) depend on the polarization of the incident light due to the orientation of microfibres. Due to the random orientation of cellulose fibres on the surface and inside the Whatman no. 1 paper structure^69^, random cellulose-derived Raman signals were more often observed for this paper type.

When paper plasmonic substrates were analysed by SEM, aggregates of spherical silver nanoparticles could be observed (Fig. 3). These aggregates of AgNPs, that are probably the result of colloidal nanoparticle aggregation upon drying inducing the formation of nanoparticle clusters with irregular structures. It seems plausible that a decreased gap size between adjacent AgNPs provides large electromagnetic field enhancements^28^. Additionally, the clusters induce a red-shifting of the LSPR band (Fig. 5), and the remaining negative charges on the Ag surface induce R6G cations to adsorb to the nanoparticles^11, 26^. This greater number of *hot spots* leads, therefore, to higher average EF values and allow the detection of lower analyte concentrations. The average EF values for AgNPs-plasmonic paper substrates, were higher than expected^70^, given their isotropic morphology, reaching an EF value of (8.4 ± 0.2) × 10^6^ and (2.1 ± 0.7) × 10^6^ in office and Whatman no. 1 papers, respectively (Fig. 6). In fact, colloids of spherical silver nanoparticles, aggregated with salt, one of the most common plasmonic SERS substrates, exhibit typically EF values in the order of 10^5^–10^7^ ^6, 71^. Thus, this paper-induced aggregation of spherical AgNPs seems to be a viable alternative to salt-induced aggregation, a classical methodology used to obtain nanostructured and SERS-active surfaces^1, 8^.

Comparing both nanoparticle’ morphologies (spherical and star shaped), AgNSs allowed in average higher EF values (Fig. 6C,D), and at lower nanoparticles densities, compared to AgNPs. Such difference can be related to the morphology of both nanoparticles. In fact, AgNSs have higher anisotropy than AgNPs, a parameter that is intrinsically related to the number of *hot spots* contributing to the overall signal. In AgNSs, *hot spots* can be found in the sharp protrusions of the stars, and between the junctions of two particles, while in AgNPs, *hot spots* are located only at interparticle spaces, originated upon cluster formation^1^. A higher number of *hot spots per* particle leads to higher intensities in the SERS spectra, and thus, the average EFs values increase. The broad LSPR produced by arms of different lengths in AgNSs colloidal solution includes the wavelength region of the excitation laser (see Fig. 5). Hence, AgNSs have a higher SERS efficiency in comparison to AgNPs, as the latter present a less broad LSPR as also reported by other authors^7, 23, 58^. It should be mentioned that nanospheres and nanorods presented in colloidal solution of AgNSs (a result from some incomplete reduction reactions) in a small amount, are also seen in the paper SERS substrate (Fig. 3B) which can contribute to the overall SERS signal. However, when comparing the number of *hot spots* present in just one nanostar, with those in a nanosphere dimer or a nanorod (two *hot spots* in each tip), is reasonable to consider that the overall observed enhancement arises mainly from the nanostars. Also, SERS measurements include the contribution of several AgNSs reducing the influence of different morphologies in plasmonic enhancements, thus leading to a low standard deviation (SD) of the EF values. As a result, this type of paper SERS substrates reveals excellent intra-well reproducibility.

**Figure 8:**
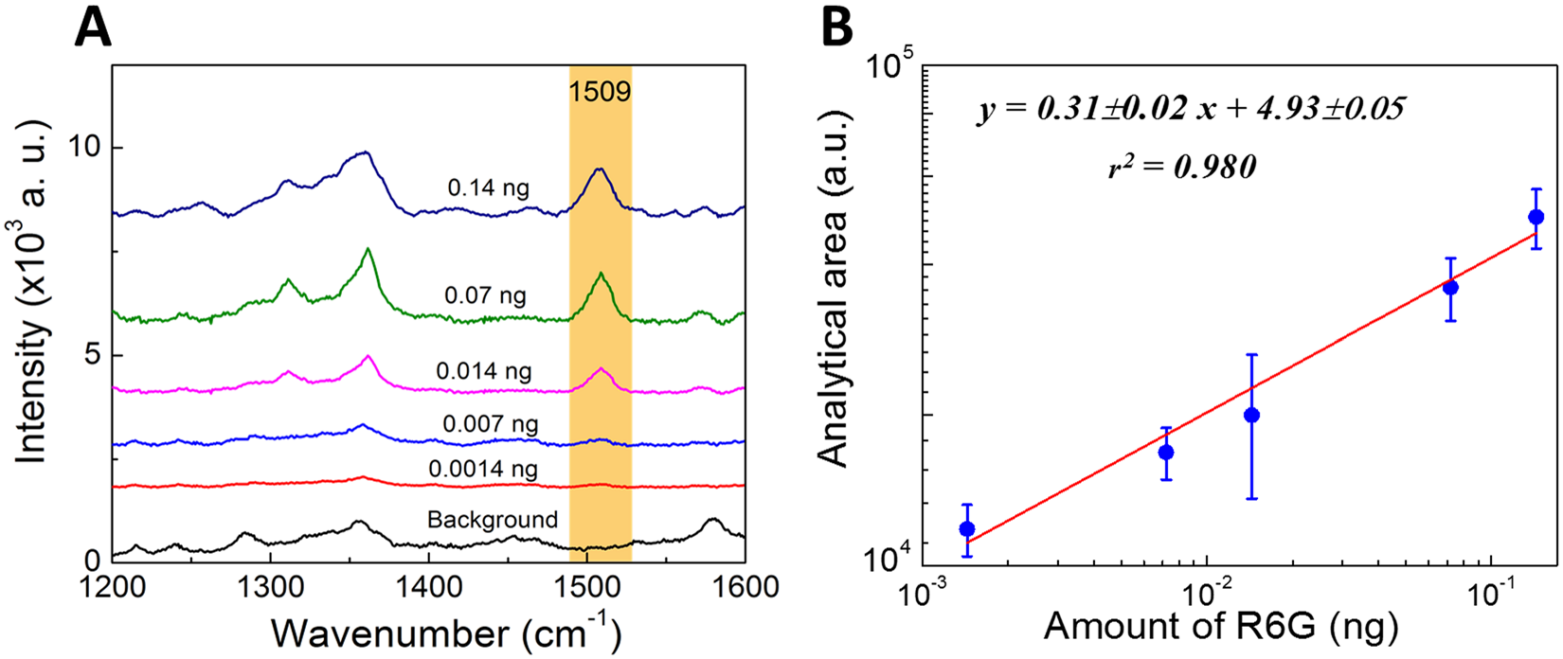
Detection sensitivity of the office paper plasmonic substrate with AgNSs. (**A**) R6G SERS spectra with 2 × 10^9^ NPs/mm^2^ of AgNSs drop-cast in office paper. Background, well without R6G, i.e., only AgNSs in office paper; amounts of R6G were varied in the range 10^−3^–10^−9^ M *i.e*. 1 × 10^3^–1 × 10^−3 ^ng (only 1 × 10^−7^–1 × 10^−9^ M *i.e*. 1 × 10^−1^–1 × 10^−3 ^ng, are represented). Areas were calculated for the 1509 cm^−1^ vibrational line (yellow band). (**B**) Correlation between logarithmic SERS band areas at 1509 cm^−1^ (due to C-C stretching vibration is highly sensitive to the amount of R6G) and logarithmic R6G amounts. Each data point represents the average value from three SERS spectra. Error bars represent the SD.

The plasmonic system revealing highest sensitivity for R6G detection and the highest average EF value (office paper with an AgNSs density of 1.7 × 10^9^ NPs/mm^2^) was further characterized. To evaluate the detection sensitivity of this plasmonic substrate, SERS spectra of different amounts of R6G from one stock solution were measured (Fig. 8).

A good correlation between the SERS 1509 cm^−1^ line areas, and the amount of R6G, was observed for concentrations ranging from 10^−3^ to 10^−9^ M (1 × 10^3 ^ng to 1 × 10^−3 ^ng). At low amounts, the band area increases linearly (equation in inset - Fig. 8) where *y* corresponds to the analytical area from the band at 1509 cm^−1^, and *x* the R6G amount. The linear correlation in the range 10^−7^ to 10^−9^ M (1 × 10^−1^ to 1 × 10^−3 ^ng) R6G was used to determine the limits of detection (LOD) and of quantification (LOQ), sensitivity (slope of the working curve), and the linearity (r^2^) from the optimized system. The LOD is defined as the lowest concentration at which a R6G SERS spectrum can be obtained. LOD was calculated by dividing three times the SD of the background signal by the slope of the calibration curve^72, 73^. Therefore, LOD could be determined as 11.4 ± 0.2 pg of R6G. The LOQ is based on the maximum acceptable relative standard deviation (RSD), commonly 10%, of a reported value^72, 73^. Dividing by the slope of the calibration curve, yields a LOQ of 34 ± 7 pg of R6G. The detection limit of AgNSs paper substrates for R6G is low, because even the spectrum of the sample for that amount had a good signal-to-noise ratio, and the main vibrational lines were still present. Considering the well area the limits of detection and quantification presented here are only slightly higher than previous works but have much less background noise^7, 9^. If we consider only the laser spot area, the limits of detection and quantification are four orders of magnitude lower than what have been reported by more sophisticated techniques such as inkjet-printing^7^.

### Reproducibility and stability of plasmonic paper substrates

**Figure 9:**
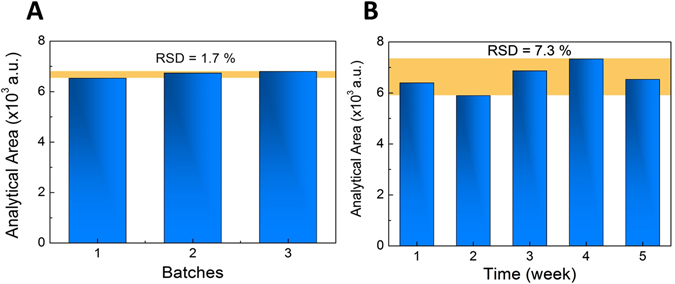
(**A**) Reproducibility of SERS substrate (office paper with AgNSs drop-casted). Area distribution of the 1509 cm^−1^ line in the spectra versus the number of batches of AgNSs colloidal solution. (**B**) Stability of SERS substrate (office paper with AgNSs drop-casted). Area distribution of the 1509 cm^−1^ line in the spectra versus stored time (5 weeks). Each data point represents the average value from three SERS spectra measured at the vicinity of each spot. The yellow region shows the relative standard deviation (RSD).

The homogeneity of SERS signals from different wells is an important parameter in evaluating the usefulness of SERS substrates, especially for its potential mass production. To test the reproducibility of the optimized SERS substrate (AgNSs substrates in office paper), nine plasmonic wells, from three independent AgNSs synthesis batches, were randomly selected and spectra were collected at room temperature for three different R6G concentrations 10^−7^, 10^−8^ and 10^−9^ M (amounts of 1 × 10^−1^, 1 × 10^−2^ and 1 × 10^−3 ^ng). Results of relative standard deviation (RSD) were calculated by variation of the 1509 cm^−1^ Raman spot-to-spot area (Fig. 9 and see Fig. S8 in Supplementary Information for all the conditions tested). It can be concluded that drop-casting of AgNSs on the office paper substrate yields uniform nanostructured surfaces over a scale of several microns, leading to a homogenous distribution of *hot spots*, and resulting in highly reproducible SERS responses (Table 1) – since the focused laser spot, has approximately 1 µm of diameter.

**Table 1:** Summary table of the RSD values regarding the reproducibility and time-stability studies.

*Amount of R6G (ng)*	*RSD (%) between batches (n = 3)*	*RSD (%) between different weeks (n = 5)*
*1*	—	10.7
*0.1*	4.4	—
*0.01*	9.6	13.7
*0.001*	1.7	7.3

In addition to the high sensitivity and good reproducibility, paper SERS substrates showed remarkable stability for field-based detection. For routine SERS analyses, an ideal substrate should be stable for a considerable amount of time. Thus, the stability of the optimized paper SERS substrate was systematically investigated over a period of 5 weeks and between measurements, it was stored at 4 °C in a desiccator. It can be clearly seen from Fig. 9, that the signals of SERS spectra after 5 weeks were very similar to the one obtained in freshly prepared substrates (see Fig. S9 in Supplementary Information for all the conditions tested). Neither a shift in the major Raman bands, nor a significant change in Raman area were observed (*data not shown*). The RSD obtained are represented in Table 1.

## Conclusions

This work presents office paper decorated with silver nanostars as an easy to fabricate, low-cost plasmonic substrate, with applications in trace analyte detection by SERS. Two types of paper (filter and office) were tested both with spherical and anisotropic (nanostars) silver nanoparticles. Whatman no. 1 paper is typically used in this type of SERS substrates, but SERS applications are scarce for office paper which, due to its structure, can advantageously concentrate nanoparticles at its surface. The highest average EF value of (3.0 ± 0.7) × 10^7^, was obtained for office paper with a density of AgNSs of 1.7 × 10^9^ NPs/mm^2^, a result in the upper limit previously described for paper substrates^6, 7^, and higher than average for paper substrates obtained by more complicated techniques, such as screen-printing with AgNPs^11^. Our plasmonic substrate presented a LOD of 11.4 ± 0.2 pg; and a LOQ of 34 ± 7 pg, for R6G.

Results indicate a model of nanoparticle distribution on paper substrates that explain their exceptional SERS performance: when nanoparticles are drop-casted from a colloidal solution onto a paper well, the paper pores provide strong capillary forces for the diffusion of nanoparticles from the colloidal solution. This allows distribution of the nanoparticles along the whole Whatman no. 1 paper thickness, while leading to nanoparticle accumulation only at the surface of office paper controlled by the swiftly drying of water. This nanoparticle uniform distribution at the office paper surface is responsible for its SERS enhanced capabilities in relation to Whatman no. 1. Likewise, the three-dimensional uneven cellulose fibre structure in office paper was responsible for the creation of a roughened silver surface potentiating the appearance of *hot spots*. These *hot spots* are usually created with spherical AgNPs by salt aggregation, so this paper-based system circumvented that aggregation step, very common in routine synthesis of SERS substrates from colloidal AgNP solutions. For AgNSs, already containing *hot spots* in their morphology, the uniform and closely distribution of AgNSs at the surface of the office paper lead to an even greater enhancement of the SERS effect by creating new *hot spots*.

Another decisive feature for the use of office paper in SERS detection is the observation that the presence of silver nanoparticles of either morphology at the office paper surface, cancels the endogenous fluorescence from paper, thus allowing sensitive SERS analysis.

Reproducibility and time stability studies proved that the simple method to produce our paper plasmonic substrates is competitive regarding others, namely on-paper silver mirror reactions. Office paper with AgNSs is a simple to obtain, robust, reproducible, stable for long-term storage between measurements and inexpensive paper plasmonic substrate, with numerous opportunities in integrating SERS with other chemical and biological multi-analytical stages such as on-site environmental monitoring and food analysis by SERS.

## Electronic supplementary material

Supplementary info
