# Supplementary material for: Office paper decorated with silver nanostars - an alternative cost effective platform for trace analyte detection by SERS
Source: Sci Rep. 2017 May 30;7:2480. doi: 10.1038/s41598-017-02484-8 (PMC5449394; doi:10.1038/s41598-017-02484-8)
Supplement: Supplementary file 1 — Supplementary info [file 41598_2017_2484_MOESM1_ESM.pdf]

Electronic Supplementary Information (ESI)

Office paper decorated with silver nanostars - an alternative cost effective platform for trace analyte detection by SERS

Maria João Oliveira[1,2], Pedro Quaresma[3], Miguel Peixoto de Almeida[3], Andreia Araújo[1], Eulália Pereira[3], Elvira Fortunato[1], Rodrigo Martins[1], Ricardo Franco[2*] and Hugo Águas[1**]

[1]CENIMAT-I3N, Departamento de Ciência dos Materiais, Faculdade de Ciências e Tecnologia, FCT, Universidade Nova de Lisboa, 2829-516 Caparica, Portugal

[2]REQUIMTE/UCIBIO, Departamento de Química, Faculdade de Ciências e Tecnologia, Universidade NOVA de Lisboa, 2829-516 Caparica, Portugal

[3]REQUIMTE/UCIBIO, Departamento de Química e Bioquímica, Faculdade de Ciências, Universidade do Porto, 4169-007 Porto, Portugal

** hma@fct.unl.pt; * ricardo.franco@fct.unl.pt

S1. Determination of the diameter and molar concentration of AgNPs by UV-Vis spectroscopy 2

S2. Determination of the diameter and concentration of AgNSs in colloidal solution using Nanoparticle Tracking Analysis 3

S3. Vibrational lines assignment for rhodamine (R6G) 5

S4. Enhancement Factor calculation 6

S5. Raman and SERS signal of rhodamine 6G 7

S6. Hydrophobic barriers on paper substrates 8

S7. Characterization of the synthesized nanoparticles 9

S8. Distribution of AgNS on Whatman no.1 and Office papers 10

S9. Office paper fluorescence elimination by silver nanoparticles 12

S10. Interference signals in SERS spectra of AgNSs 13

S11. Reproducibility between different AgNSs synthesis batches 15

S12. Time stability of the plasmonic paper substrate 16

S13. References 17

S1. Determination of the diameter and molar concentration of AgNPs by UV-Vis spectroscopy

To calculate the average diameter and the molar concentration of AgNPs the Paramelle *et al.*[1] method was used. Previously, Haiss *et al.*[2] reported a similar method to calculating these parameters for spherical AuNPs coated with citrate using the respective absorption spectrum. First, the average diameter of AgNPs is determined by relating this parameter (x) with maximum absorbance (y) by the following equation:

$$y = 397 + 5.85 \times 10^{-2} x^{2} \qquad \text{Equation (1)}$$

After inferring the average diameter of the AgNPs (≈23 nm), it is possible to estimate the molar extinction coefficient thereof by Equation 2:

$$y = -1.493 \times 10^{9} + 6.984 \times 10^{8} e^{0.104x} \qquad \text{Equation (2)}$$

Achieving $\varepsilon_{402\ nm}$ value of ≈6.21×109 $M^{-1}cm^{-1}$. Finally, using Lambert-Beer's law (Equation 3), where b, is the optical path length (1 cm), and c is the molar concentration, the concentration of the AgNPs solution is approximately 0.66 nM.

$$A = \varepsilon_{402\ \mathrm{nm}}\, bc \qquad \text{Equation (3)}$$

Bastús *et al.* method provides an improved procedure for the AgNPs synthesis over the method of Lee and Meisel.[3,4] Well distributed AgNPs with a desired diameter were obtained. Moreover, the lack of use of strong surfactants promotes the accessibility of the surface of the NPs, a crucial factor for efficient SERS.[5]

S2. Determination of the diameter and concentration of AgNSs in colloidal solution using Nanoparticle Tracking Analysis

Nanoparticle Tracking Analysis (NTA) methodology was first described in 2006 by Malloy and Carr[6]. The technique is based on the analysis of the Brownian motion of nanoparticles in colloidal solution. A laser beam goes through the sample chamber and the light scattered by the nanoparticles that cross the laser path is captured by a microscope-like assembly that directs the light to a digital image detector. A set of short videos (*e.g.* 60 seconds) is recorded and the light scattered by individual particles (bright circular shapes) can be easily seen on it. Video analysis of the path of the individual particles, allows the calculation of the mean squared displacement (MSD) for each particle. From these MSD values, diffusion coefficient (D) can be obtained and, related to the hydrodynamic radius by the Stokes-Einstein equation:

$$D = \frac{K_B T}{6\pi\eta r} \qquad \text{Equation (4)}$$

where k_B is the Boltzmann's constant, T the temperature, η the solvent viscosity, and r is the hydrodynamic radius.

At the same time, the number of nanoparticles *per* volume can be calculated, since the volume of the analysed solution is known, and the number of particles is counted by the software.

A typical NTA analysis for the AgNSs (triplicate samples) gives a mean hydrodynamic diameter = 177.6 ±3.3 nm, mode = 192.0±20.4 nm, SD = 59.2±1.9 nm. Only 10% of the particles have hydrodynamic diameters lower than 109.1±4.2 nm or higher than 254.3 ±4.1 nm.

The synthesis of anisometric nanoparticles was confirmed also by TEM (Figure S 1), proving the homogeneity of the sample relative to the star content. Other morphologies are detected in the samples, namely irregular spheres and rods, but these consistently represent less than 10% of the total nanoparticles.

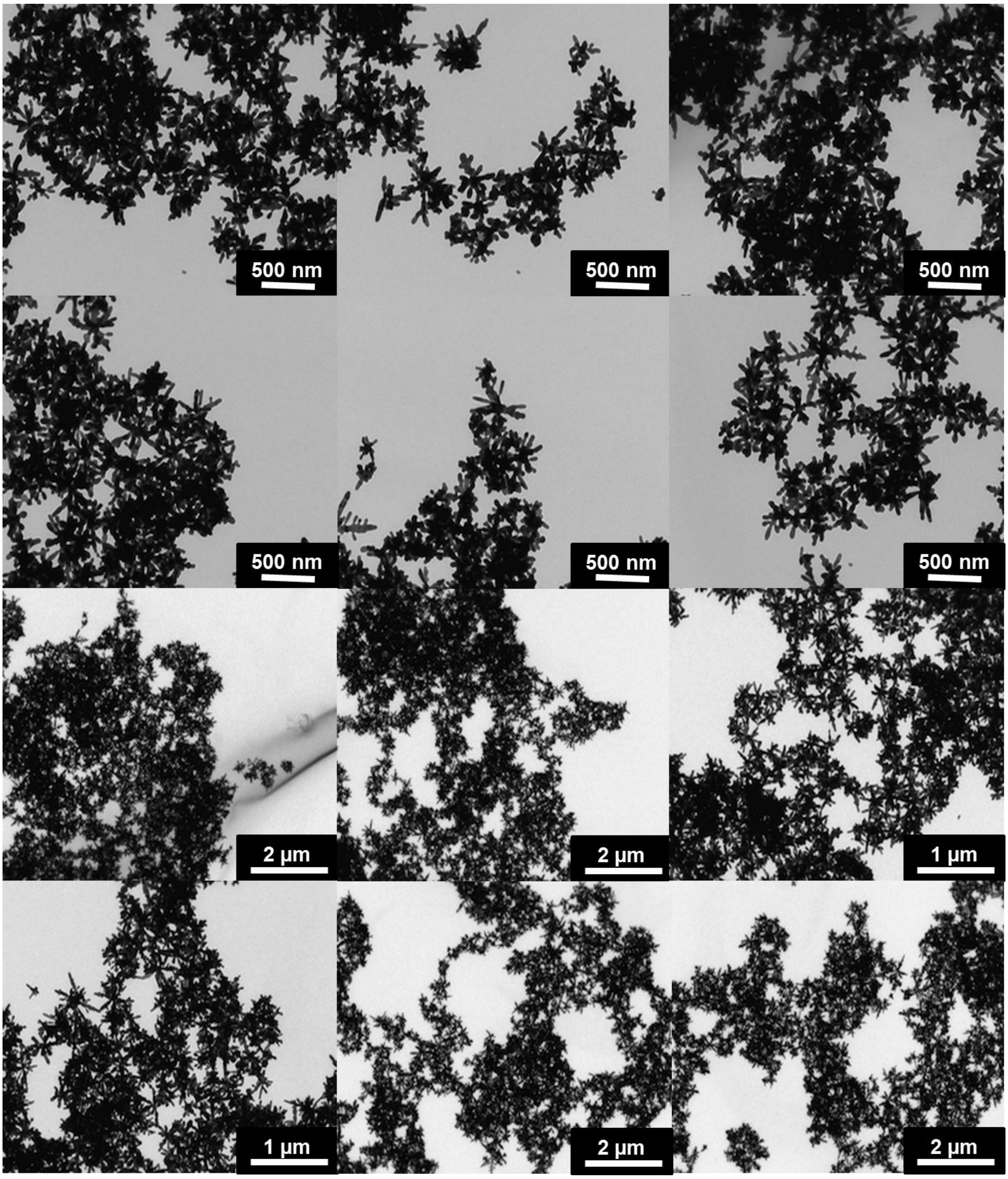

Figure S 1 – Several TEM micrographs of AgNSs solution to evaluate the morphologies of the particles. AgNSs are morphologically characterized by several tips protruding from a central core with a tip-to-tip length around 200 nm.

S3. Vibrational lines assignment for rhodamine (R6G)

Vibrational lines assignments for R6G are presented in Table S1. The areas of the vibrational Raman lines at 1360 cm^{-1} and 1509 cm^{-1} were used to calculate spectral intensity as usually reported in the literature [7,8].

Table S 1 - Vibrational lines assignments for R6G and correspondences to the observed Raman lines.[9,10]

**Experimentally observed bands (cm^{-1})**		**Strength**	**Assignment**
**Raman**	**SERS**		
	1030	vw	
	1127	m	in plane C–H bending
1183	1182	m	C–H bending, N–H bending, in plane xanthene ring deformation
1310	1310	s	In plane xanthene ring breathing, N–H bending, CH_2 waggling
1362	1361	s	Xanthene ring stretching, in plane C–H bending
	1454	w	COH bending
1509	1509	vs	Aromatic C–C stretching, xanthene ring stretching, C–N stretching, C–H bending, N–H bending
1574	1574	m	Xanthene ring stretching, in plane N–H bending
1597	1597	w	
1649	1649	s	Xanthene ring stretching, in plane C–H bending

Abbreviations: vw: very weak; w: weak; m: medium; s: strong; vs: very strong.

S4. Enhancement Factor calculation

The average SERS enhancement factor (EF) is calculated according to the equation:[11]

$$EF = \frac{I_{SERS}}{I_{Raman}} \times \frac{N_{Raman}}{N_{SERS}}$$

Equation (5)

Where, I_{SERS} is the SERS intensity of a particular Raman line of the analyte (in this case R6G) and I_{Raman} is the normal (not enhanced) Raman intensity of the R6G measured over a non-plasmonic reference substrate. The Raman signal of the reference is, in most cases, too weak to be detected when measuring small analyte concentrations on the surface. Therefore, the analyte concentration applied to measure the reference Raman spectra is usually higher than that used in the SERS spectra, so a correction factor (N_{Raman} /N_{SERS}) is introduced in the EF expression to take that into account. In the present measurements, N_{SERS} corresponds to the estimated number of molecules contributing to the SERS signal, while N_{Raman} is the number of molecules contributing to the reference Raman signal (from non-SERS surface). Both values are related with the available area of the SERS substrate and the laser spot focus. They are determined by the relation:

$$N_{SERS} = \eta \times N_A \times V \times C_{SERS} \times \frac{A_{laser}}{A_{SERS}} \; ; \; N_{Raman} = N_A \times V \times C_{Raman} \times \frac{A_{laser}}{A_{Raman}}$$

Equation (6) and (7)

Where N_A is the Avogadro number, V is total volume of solution spread on the substrate (2 µL), A_{Laser} is the area of the laser spot (8.32×10^{-7} mm^2), A_{SERS} and A_{Raman} are the total area of the SERS and non-SERS reference substrate respectively, covered by the drop of analyte solution. C_{SERS} and C_{Raman} are the concentrations of analyte applied over the SERS and non-SERS substrates, respectively. Since the same volume of solution was applied in both substrates, $A_{SERS} \approx A_{Raman}$ =3.14 mm^2. The dimensionless adsorption factor, η, is taken to be 0.5 for the plasmonic paper SERS substrates. This adsorption factor is based in Langmuir isotherm, and can be expressed in the form: η=1/(1+Kc_0); where c_0 is the initial concentration of the analyte at saturation level and K the equilibrium binding constant. For a more precise determination of the EF, the calculations of both the I_{SERS} and I_{Raman} intensities consider the area under the Raman vibrational bands at 1360 and 1509 cm^{-1}.

S5. Raman and SERS signal of rhodamine 6G

Figure S 2 presents the Raman spectrum of a 10^{-3} M R6G aqueous solution deposited on a bare glass plate and the SERS spectrum of a 10^{-6} M R6G aqueous solution deposited onto AgNSs on office paper using laser 632.8 nm laser excitation.

These spectra are in good agreement with respect to both the frequencies and the relative intensities with those reported in the literature. The vibrational bands energies are highly conserved between Raman and SERS, which is indicative that there is an electrostatic interaction between R6G and the Ag surface [12].

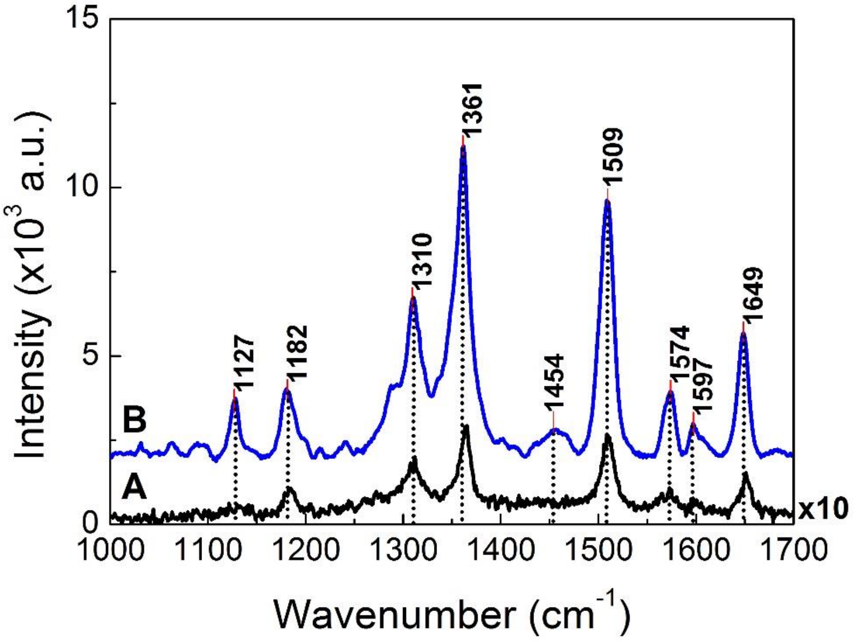

Figure S 2 - Raman and SERS spectra of R6G aqueous solutions obtained with a 632.8 nm laser. (A) Raman spectrum of 10^{-3} M (1440 ng) R6G (×10 magnified) and (B) SERS spectrum of 10^{-6} M (1.44 ng) R6G deposited on AgNSs on office paper. Vibrational energies indicated in the lines labels, are as reported in the literature for R6G.

S6. Hydrophobic barriers on paper substrates

The fabrication of paper SERS substrates involved the *Lab-on-paper* technology, based on printing hydrophobic wax patterns and barriers on paper [13]. These patterns were then advantageously used to support the metal nanoparticles deposited by drop-casting method. Due to the confinement, the diffusion outside the wells is prevented which allowed a uniform distribution and a higher concentration of the nanoparticles (Figure S 3). In addition, the barriers prevent contamination of adjacent samples, even in the more hydrophobic office paper [14], allowing multiple assays.

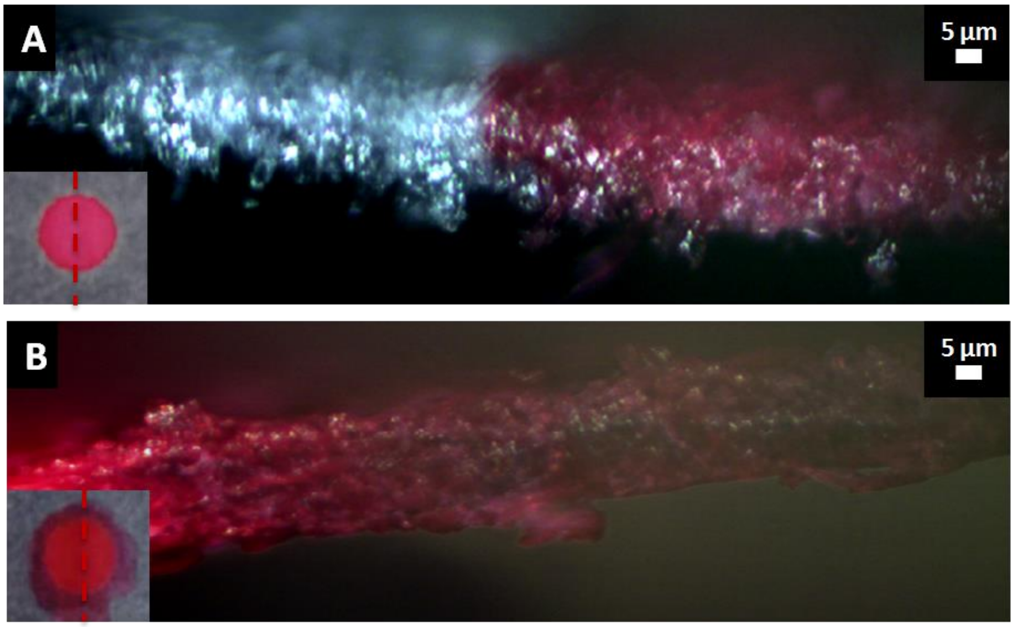

Figure S 3 - Optical microscopic images of paper cross sections (red dash line in *inset*), illustrating the role of the hydrophobic barriers formed by wax diffusion. The pink colour shown on the paper's sample is R6G. Wax printing is only at the surface of the paper, and wells are created by a heating step that promotes wax diffusion. Paper with (A), and without (B) wax diffusion.

S7. Characterization of the synthesized nanoparticles

Solutions of spherical AgNPs presented a yellow colour and its UV-Vis spectrum showed a band centred at around 400 nm, which was assigned to their LSPR (Figure S 4 - Line A). The width and position of this band can be used to determine the average size and molar concentration of nanoparticles. Their optical properties depend on its size and morphology, verifying the red-shift phenomenon, related to a decrease in the plasmonic frequency.[1,2] The Paramelle *et al.* method was used to calculate the average diameter (≈23 nm) and the molar concentration of AgNPs.[1]

Spherical AgNPs were aggregated in solution by adding 50 mM of NaCl. This process could be followed by UV/vis spectroscopy, by a red-shift (to ≈745 nm) and broadening of the LSPR band Figure S 4 - Line B). This band (≈745 nm) presumably indicates nanoparticle dimers and higher aggregates.[15]

Silver nanostars (AgNSs) give rise to a spectrum displaying one strong peak at ≈379 nm (Figure S 4 - line C). At longer wavelengths, an extinction background arises from very different LSPR bands in the suspension giving a wide range of wavelengths in the visible and near-IR. Wavelengths at the near-IR region with a broad width are characteristic of the structures with sharper corners that lead to higher enhancements. The several distinct LSPR bands derive from different morphological shapes of the AgNSs, with different number of arms and varying tip sharpness.[16] Silver NSs with larger vertex angles or a higher number of arms, are probably involved in the more modest multi-pole resonance peak and narrower bandwidth at lower wavelengths (blue-shift of resonant wavelength).[16–18]

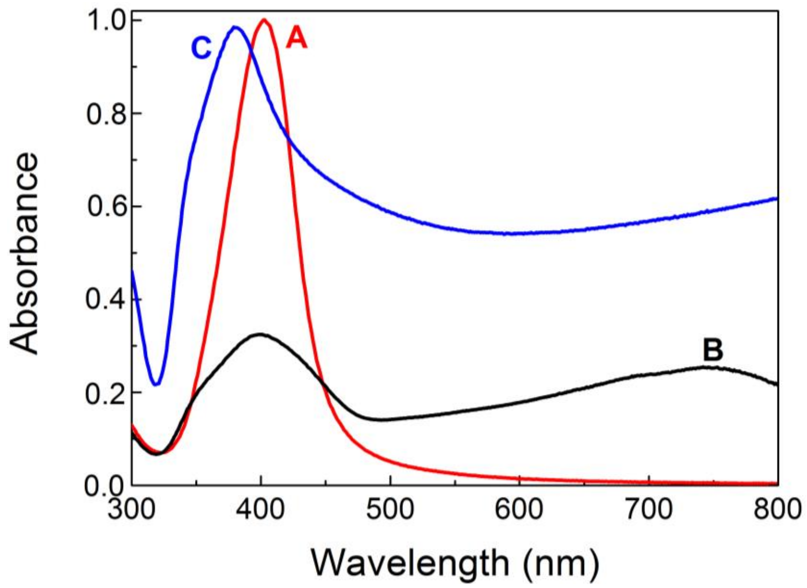

Figure S 4 - UV-vis spectra of non-aggregated spherical AgNPs (A - red) with λmax= 402 nm; aggregated spherical AgNPs (B - black) with λmax= 402 and 743 nm; and AgNSs (C - blue) with λmax= 379 nm.

S8. Distribution of AgNS on Whatman no.1 and Office papers

AgNPs and AgNSs colloidal solutions (10 µL) were drop-casted in the wells forming the paper SERS substrate. The concentrations of AgNPs and AgNSs added to the 2 mm wells were 0.22, 0.44, 0.88, 1.76, 3.52, 7.04 nM. By knowing the concentrations and volumes dropped in the wells with a defined area it is possible to determine the corresponding amounts of nanoparticles *per* area of the well (NPs/mm^2).

SEM analysis of the wells with increasing concentration of colloidal solution showed that, as expected, increasing the concentration of nanoparticles improves the coverage of the surfaces regardless of the type of paper. For the same number of applied nanoparticles, office paper shows a much larger amount of nanoparticles on the surface, when compared with Whatman no.1 paper.

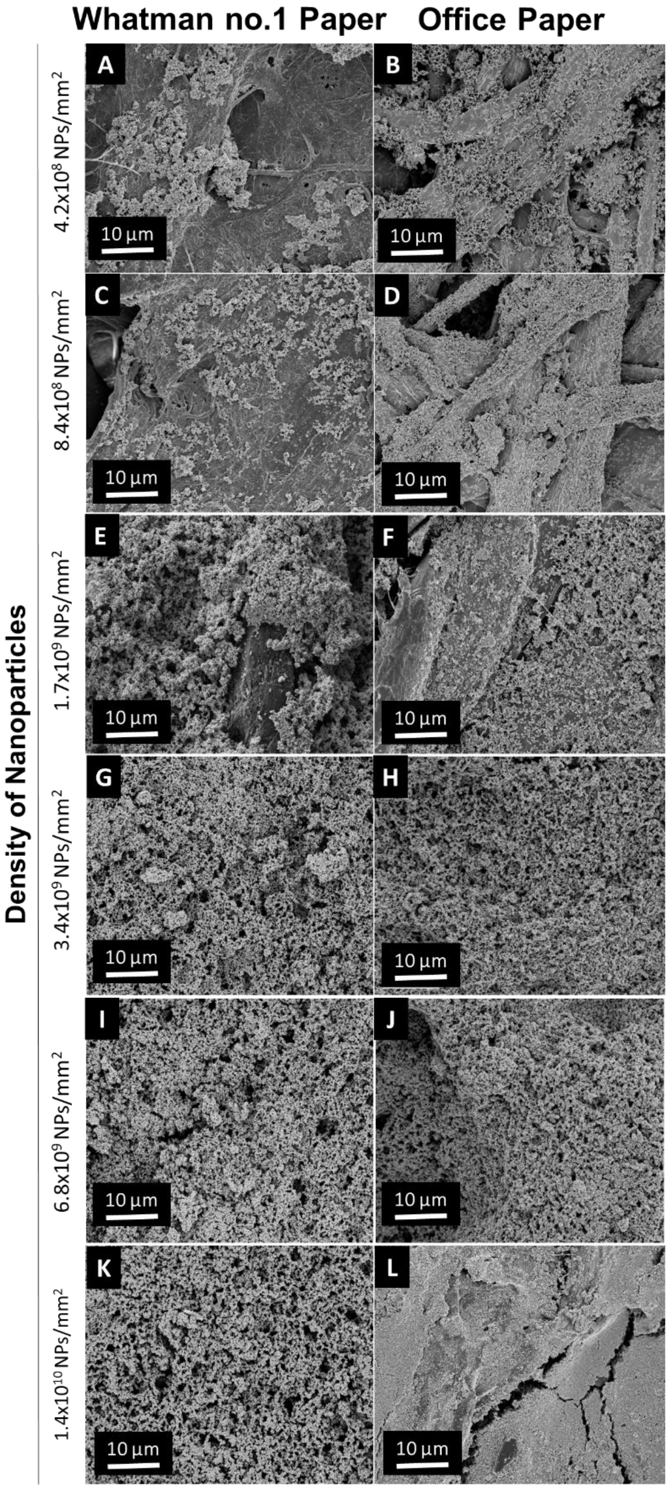

Figure S 5 - SEM images of AgNSs in the two types of papers: (1) Whatman no.1 and (2) office paper. From the left to the right of the figure, the amount of AgNSs added onto the paper well increases from (A and B) $4.2x10^8$ NPs/mm^2; (C and D) $8.4x10^8$ NPs/mm^2; (E and F) $1.7x10^9$ NPs/mm^2; (G and H) $3.4x10^9$ NPs/mm^2; (I and J) $6.8x10^9$ NPs/mm^2; (K and L) $1.4x10^{10}$ NPs/mm^2. More NPs were observed in the paper surface for higher volumes added, regardless of the type of paper. However, for office paper, the amount of AgNSs that is necessary to cover the same surface area of the paper, is much lower than for Whatman no.1 paper.

S9. Office paper fluorescence elimination by silver nanoparticles

Office paper samples showed fluorescence emission with excitation under the Raman laser (red beam). However, this fluorescence was eliminated after the addition of silver nanoparticles (Figure S 6). This can be explained by the fact that in the one hand, metal nanoparticles tend to quench fluorescence emission signals. On the other hand, the deposited metal nanoparticles on the surface can shield any fluorescence emission from the paper material [19].

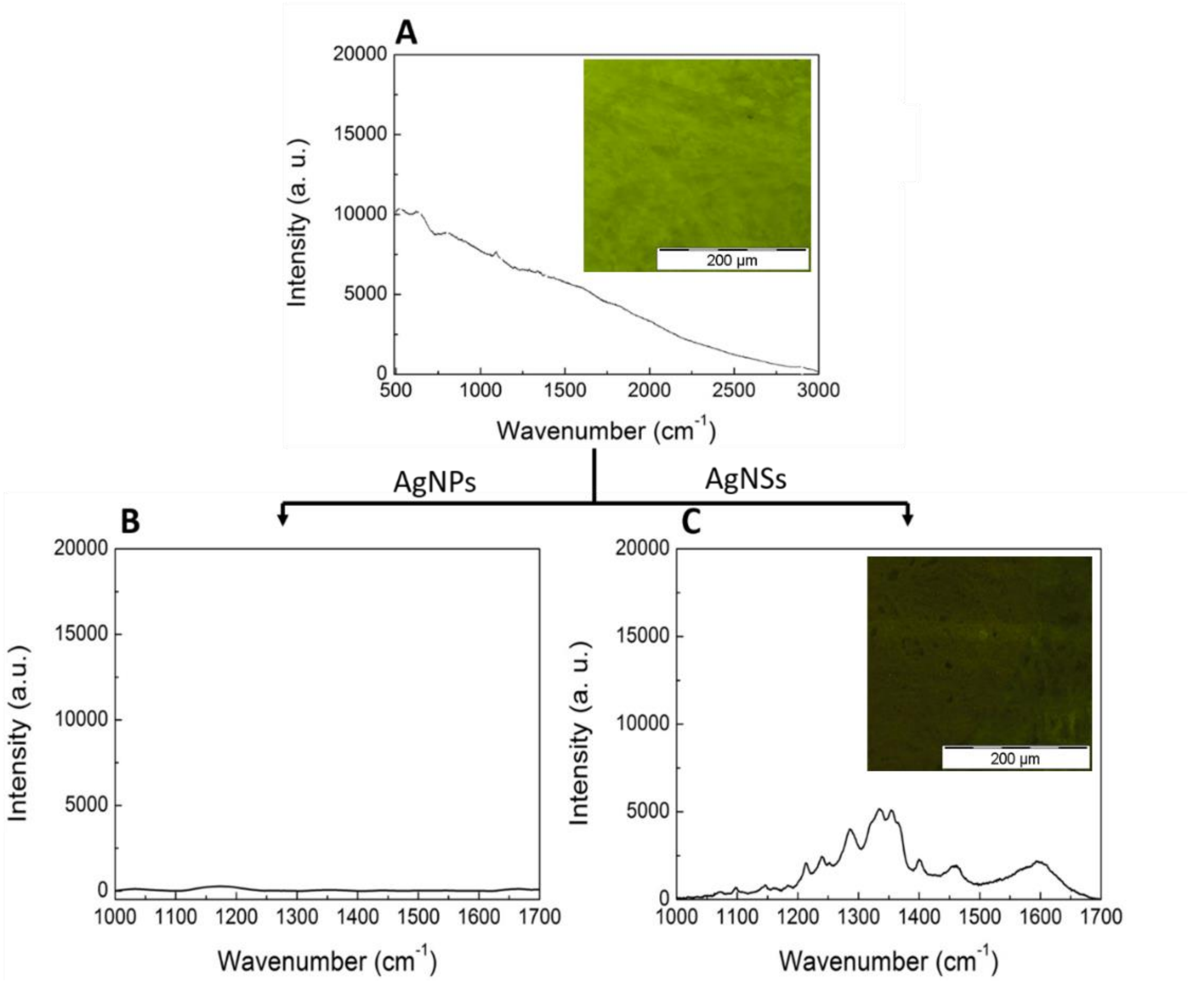

Figure S 6 - SERS spectra of office paper alone (A); after addition of AgNPs (B); and after addition of AgNSs (C). The noticeable fluorescence background on office paper alone, disappears after addition of both types of silver nanoparticles. The Raman lines observed for AgNSs (C), are assigned to its capping-agent, citrate (Figure S 7). The insets in A and C refer to fluorescence images of paper before and after AgNSs addition.

S10. Interference signals in SERS spectra of AgNSs

Contaminants even in a lower concentration may be present in the sample. They can be selectively enhanced through a resonance Raman scattering mechanism, overlapping the bands of the adsorbate under study.[20] Therefore, the positions of the vibrational lines were evaluated and confronted with components that were known to be present on the paper SERS substrate: (1) paper; and (2) reagents used in the NPs synthesis, such as hydroxylamine and sodium citrate. The spectra of paper alone (*data not shown*) and citrate (Figure S 7 - A) were analysed according to the standard Raman spectrum. The Raman data of citrate oxidation products (acetonedicarboxylic and acetoacetic acid) and hydroxylamine were obtained from the literature.[20,21] The SERS signal of the residual nitrate (NO_3^-) from the $AgNO_3$ (the precursor for NPs synthesis) was also compared to the literature data. [22] However, no Raman lines from NO_3^- could be detected.

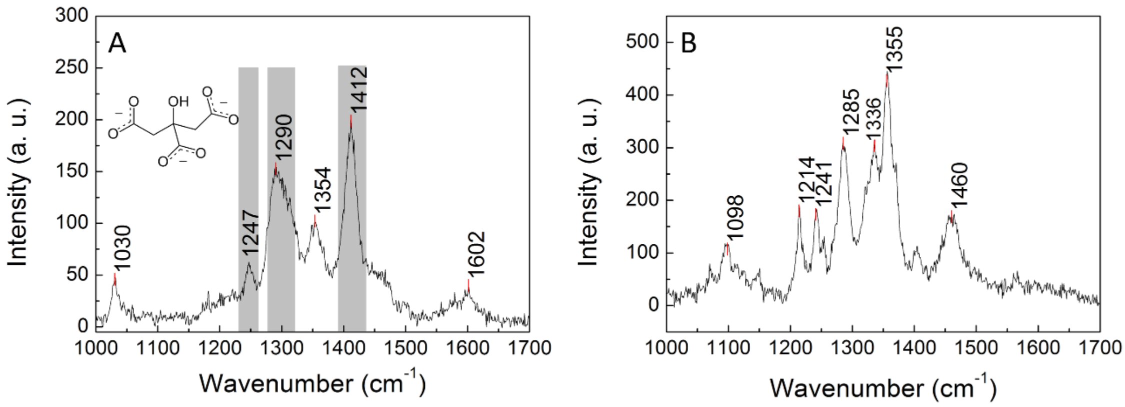

Figure S 7 - Raman spectrum of citrate. The regions representative of citrate bonding vibrations are in grey colour. On the top of the grey area are indicated the vibrational modes. (A). The SERS spectrum of AgNSs deposited on office paper presents Raman lines that are similar present in Raman spectrum of citrate.

Table S 2 - Vibrational lines assignments for citrate and correspondences to the observed Raman lines. [22,23]

Experimentally observed bands (cm^{-1})				Assignment
**Citrate**	**Strength**	**Anomalous bands**	**Strength**	
1030	w	-	-	
-	-	1098	w	
-	-	1214	m	CO stretching mode from tertiary alcohol
1247<br>1290	w<br>s	1241<br>1285	m<br>s	Carboxylate deformations (COO)
-	-	1336	vs	
1354	m	1355	vs	
1412	vs	1414	vw	Carboxylate symmetric stretching mode from citrate (COO)
-	-	1460	s	
1602	vw	-	-	

The bands in Raman spectrum of citrate (Figure S 7), are consistent with published values.[22,24] The slight deviations between the two spectra may be indicative of interactions between the citrate molecules and the Ag surface.[24] Nevertheless, SERS spectra of the observed interfering bands can be clearly attributed to citrate, since they correlate very well with the Raman spectrum of citrate in aqueous solution. In fact, Yaffe *et al.* found that in a salt-aggregated AgNP colloid, the band patterns for the citrate did not disappear after addition of the analyte.[24] The bands related to the appearance of citrate oxidation products were not identifiable, hence, it is possible that they do not interfere in R6G SERS spectra.[25] The presence of interferences in paper SERS substrates underscores the importance of the interpretation of the spectra profiles, even before the addition of the analyte.

S11. Reproducibility between different AgNSs synthesis batches

To test the reproducibility of AgNSs substrates in office paper, nine plasmonic wells, from three independent AgNSs synthesis batches, were randomly selected and spectra were collected at room temperature for three different R6G concentrations 10^{-7}, 10^{-8} and 10^{-9} M (amounts of 1×10^{-1}, 1×10^{-2} and 1×10^{-3} ng). Results of relative standard deviation (RSD) were calculated by variation of the 1509 cm^{-1} Raman spot-to-spot area (Figure S 8). It can be concluded that drop-casting of AgNSs on the office paper substrate yields uniform nanostructured surfaces over a scale of several microns, leading to a homogenous distribution of *hot spots*, and resulting in highly reproducible SERS responses.

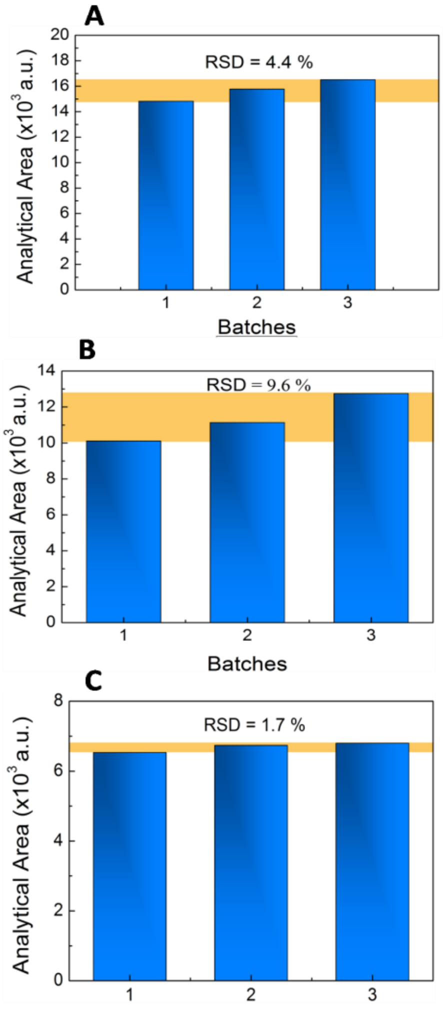

Figure S 8 - Reproducibility of SERS substrate (office paper with AgNSs drop-casted). Area distribution of the 1509 cm^{-1} band in the spectra *versus* the number of batches of AgNSs colloidal solution. Each data point represents the average value from three SERS spectra measured at the vicinity of each spot. The yellow region show the relative standard deviation (RSD) for different R6G concentrations: (A) 10^{-7} M (RSD = 4.4 %); (B) 10^{-8} M (RSD = 9.6 %) and (C) 10^{-9} M (RSD = 1.7 %).

S12. Time stability of the plasmonic paper substrate

The stability of the optimized paper SERS substrate was systematically investigated over a period of 5 weeks and between measurements, it was stored at 4 ^{o}C in a desiccator. The spectra were collected at room temperature for three different R6G concentrations 10^{-6}, 10^{-8} and 10^{-9} M (amounts of 1×10^{-1}, 1×10^{-2} and 1×10^{-3} ng). Results of RSD were calculated by variation of the 1509 cm^{-1} Raman spot-to-spot area (Figure S 9). The signals of SERS spectra after 5 weeks were very similar to the one obtained in freshly prepared substrates.

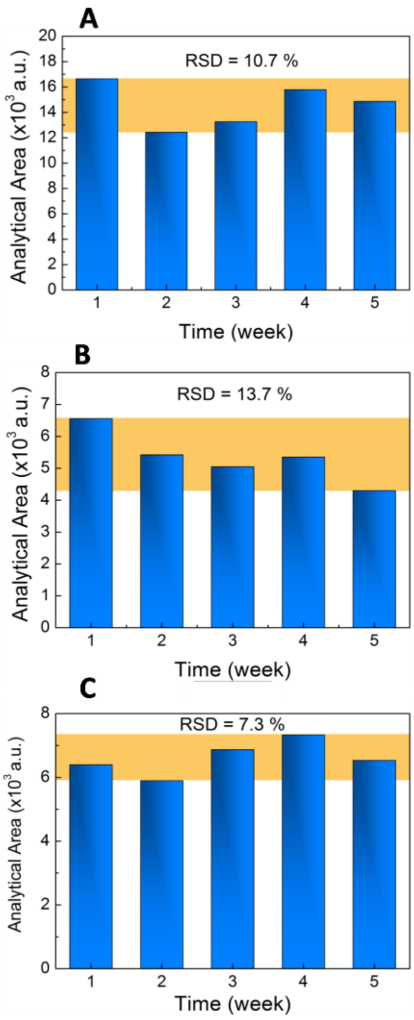

Figure S 9 - Stability of SERS substrate (office paper with AgNSs drop-casted). Area distribution of the 1509 cm^{-1} band in the spectra versus stored time (5 weeks). Each data point represents the average value from three SERS spectra measured at the vicinity of each spot. The yellow region show the RSD for different R6G concentrations: (A) 10^{-6} M (RSD = 10.7 %); (B) 10^{-8} M (RSD = 13.7 %) and (C) 10^{-9} M (RSD = 7.3 %).

S13. References

1. Paramelle, D. *et al.* Rapid method to estimate the concentration of citrate capped silver nanoparticles from UV-visible light spectra. *Analyst* **139,** 4855–4861 (2014).

2. Haiss, W., Thanh, N. T. K., Aveyard, J. & Fernig, D. G. Determination of Size and Concentration of Gold Nanoparticles from UV - Vis Spectra. **79,** 4215–4221 (2007).

3. Bastús, N. G., Merkoçi, F., Piella, J. & Puntes, V. Synthesis of Highly Monodisperse Citrate-Stabilized Silver Nanoparticles of up to 200 nm: Kinetic Control and Catalytic Properties. *Chem. Mater.* **26,** 2836–2846 (2014).

4. Lee, P. C. & Meisel, D. Adsorption and surface-enhanced Raman of dyes on silver and gold sols. *J.Phys.Chem.* **86,** 3391–3395 (1982).

5. Tao, A. R., Habas, S. & Yang, P. Shape control of colloidal metal nanocrystals. *Small* **4,** 310–325 (2008).

6. Malloy, A. & Carr, B. Nanoparticle tracking analysis - The HaloTM System. *Part. Part. Syst. Charact.* **23,** 197–204 (2006).

7. Qu, L.-L. *et al.* Fabrication of bimetallic microfluidic surface-enhanced Raman scattering sensors on paper by screen printing. *Anal. Chim. Acta* **792,** 86–92 (2013).

8. Yu, W. W. & White, I. M. A simple filter-based approach to surface enhanced Raman spectroscopy for trace chemical detection. *Analyst* **137,** 1168 (2012).

9. Jensen, L. & Schatz, G. C. Resonance Raman scattering of rhodamine 6G as calculated using time-dependent density functional theory. *J. Phys. Chem. A* **110,** 5973–5977 (2006).

10. Nagashree, K. L., Lavanya, R., Kavitha, C., Narayanan, N. S. V & Sampath, S. Spontaneous formation of branched nanochains from room temperature molten amides: visible and near-IR active, SERS substrates for non-fluorescent and fluorescent analytes. *Rsc Adv.* **3,** 8356–8364 (2013).

11. Mohiddon, M. A., Sangani, L. D. V. & Krishna, M. G. Scanning near field optical microscopy of gold nano-disc arrays fabricated by electron beam lithography and their application as surface enhanced Raman scattering substrates. *Chem. Phys. Lett.* **588,** 160–166 (2013).

12. Kleinman, S. L. *et al.* Structure enhancement factor relationships in single gold nanoantennas by surface-enhanced raman excitation spectroscopy. *J. Am. Chem. Soc.* **135,** 301–308 (2013).

13. Zhao, W. & van der Berg, A. Lab on paper. *Lab Chip* **8,** 1988–1991 (2008).

14. Costa, M. N. *et al.* A low cost, safe, disposable, rapid and self-sustainable paper-based platform for diagnostic testing: lab-on-paper. *Nanotechnology* **25,** 94006 (2014).

15. Bhatia, P. *et al.* Onset of Intense Surface Enhanced Raman Scattering and Aggregation in the Au @ Ag System. *J. Spectrosc.* **2015,** 11 (2015).

16. Ma, W. Y. *et al.* A numerical investigation of the effect of vertex geometry on localized surface plasmon resonance of nanostructures. *Opt. Express* **18,** 843–853 (2010).

17. Xu, H., Aizpurua, J., Kall, M. & Apell, P. Electromagnetic contributions to single-molecule sensitivity in surface-enhanced raman scattering. *Phys. Rev. E. Stat. Phys. Plasmas. Fluids. Relat. Interdiscip. Topics* **62,** 4318–24 (2000).

18. Garcia-Leis, A., Garcia-Ramos, J. V. & Sanchez-Cortes, S. Silver nanostars with high SERS performance. *J. Phys. Chem. C* **117,** 7791–7795 (2013).

19. Peixoto de Almeida, M. *et al.* in *Comprehensive Analytical Chemistry* **66,** 529–567 (2014).

20. Sánchez- Cortés, S. & García- Ramos, J. V. Anomalous Raman bands appearing in surface- enhanced Raman spectra. *J. Raman Spectrosc.* **29,** 365–371 (1998).

21. Krishnan, R. S. & Balasubramanian, K. Spectrum Hydrochloride of Hydroxylamine. 285–292 (1964).

22. Bell, S. E. J., Sirimuthu, N. M. S. & Colloids, C. S. Surface-Enhanced Raman Spectroscopy as a Probe of Competitive Binding by Anions to Citrate-Reduced Silver Colloids Surface-Enhanced Raman Spectroscopy as a Probe of Competitive Binding by Anions to. *Society* **109,** 7405–7410 (2005).

23. PubChem Compound Database. Chemical Structure of Citrate (CID=31348). *National Center for Biotechnology Information* at <http://pubchem.ncbi.nlm.nih.gov/compound/citrate#section=Top>

24. Yaffe, N. R. & Blanch, E. W. Effects and anomalies that can occur in SERS spectra of biological molecules when using a wide range of aggregating agents for hydroxylamine-reduced and citrate-reduced silver colloids. *Vib. Spectrosc.* **48,** 196–201 (2008).

25. Tada, H., Bronkema, J. & Bell, a T. Application of in situ surface-enhanced Raman spectroscopy (SERS) to the study of citrate oxidation on silica-supported silver nanoparticles. *Catal. Letters* **92,** 93–99 (2004).
